# Motion Compensation in Pulmonary Fluorescence Lifetime Imaging: An Image Processing Pipeline for Artefact Reduction and Clinical Precision

**DOI:** 10.1109/OJEMB.2025.3558620

**Published:** 2025-04-08

**Authors:** Tarek Haloubi, Spencer Angus Thomas, Catherine Hines, Kevin Dhaliwal, James R. Hopgood

**Affiliations:** Institute for Imaging, Data and Communications, School of EngineeringUniversity of Edinburgh 3124 EH9 3FG Edinburgh U.K.; National Physical Laboratory 9917 TW11 0LW Teddington U.K.; GlaxoSmithKline 48121 Collegeville PA 19426 USA; Centre for Inflammation ResearchUniversity of Edinburgh 3124 EH16 4TJ Edinburgh U.K.

**Keywords:** Fluorescence lifetime imaging, medical image registration, motion compensation, optical endomicroscopy

## Abstract

*Goal:* This study introduces Temporal Reliability and Accuracy via Correlation Enhanced Registration (TRACER), a novel image processing pipeline that addresses motion artefacts in real-time Fluorescence Lifetime Imaging (FLIm) data for in-vivo pulmonary Optical Endomicroscopy (OEM). Its primary objective is to improve the accuracy and reliability of FLIm image sequences. *Methods:* The proposed TRACER pipeline comprises a comprehensive sequence of pre-processing steps and a novel registration approach. This includes the removal of uninformative frames and motion characterisation through dense optical flow, followed by a tracking-based Normalised Cross Correlation image registration method leveraging Channel and Spatial Reliability Tracker for precise alignment. *Results:* The complete TRACER pipeline delivers significant performance improvements, with 20% to 30% enhancement across different metrics for all tested registration methods. In particular, the unique TRACER registration approach outperforms state-of-the-art methods in image registration performance and achieves an order-of-magnitude faster runtime than the next best-performing approach. *Conclusion:* By addressing motion artefacts through its integrated pre-processing and novel registration strategy, TRACER offers a robust solution that ensures improved image quality and real-time feasibility for FLIm data processing in *in-vivo* pulmonary OEM.

## I. Introduction

Fibre-based OEM imaging has become a prominent medical imaging platform, facilitated by advancements in miniaturised, flexible fibre-optic endoscopes [1], [2]. The application of FLIm has grown significantly in clinical research, enabling real-time diagnostics, robotic surgery, and drug discovery [1], [3]. Specifically, OEM imaging shows great potential for exploring the lung's alveolar space, an area difficult to assess with traditional imaging techniques [2]. In this approach, laser-focused light is delivered via a miniaturised fibre-bundle to the alveolar space to excite tissue, stimulating fluorophore molecule fluorescence [4]. A detector captures the emitted photons, and the temporal responses across multiple optical wavelengths are used to generate FLIm images by characterising fluorescence lifetimes from the decay profiles, using the rapid lifetime determination (RLD) algorithm or more advanced methods like robust RLD or fit-flexible algorithms [5], [6]. The system used in this study captures both fluorescence intensity and lifetime images of a biological setup employed in clinical experiments, closely approximating the system's intended use in human lungs [7].

Fluorescence intensity reflects the emission process following photon energy absorption from an external source, causing an energy transition from ground to excited states. In contrast, fluorescence lifetime represents the average time between excitation and the return to the ground state. FLIm is an important bio-imaging method for studying molecular and cellular interactions [8]. Recent studies by Craven et al. [9] and Duncan et al. [7] demonstrate the potential of OEM-FLIm in detecting immune responses within the pulmonary system, highlighting its use for real-time disease monitoring.

**Fig. 1.**
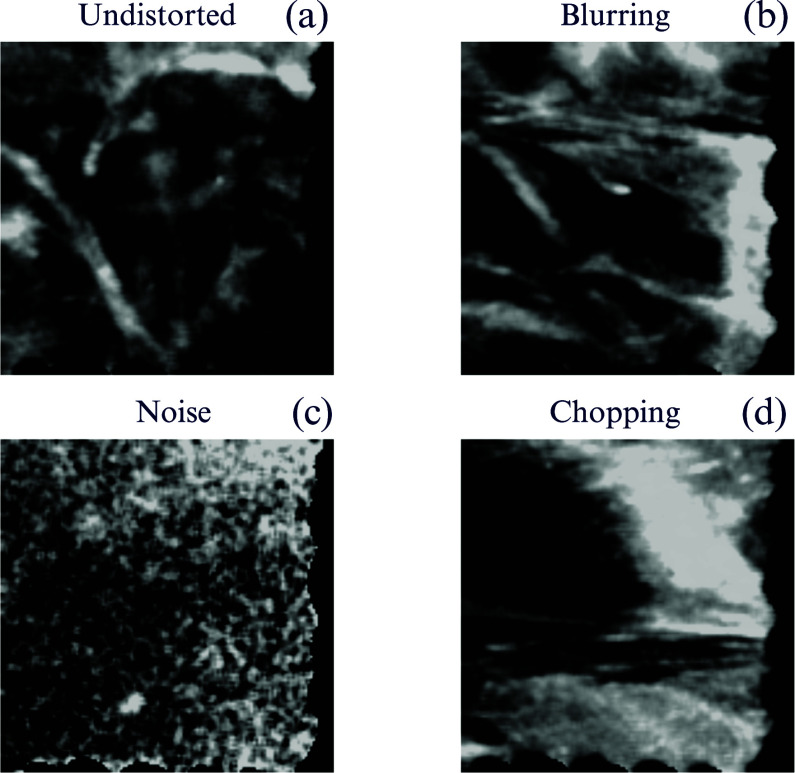
Examples of artefacts observed in the FLIm imaging data. (a) Undistorted image, (b) blurred image, (c) corrupted image, (d) distorted image.

However, despite these advancements, fibre-based FLIm data often display structural abnormalities, as shown in Fig. 1. These abnormalities manifest as blurring or chopping, where the image is degraded by random variations in colour or brightness, as illustrated in Fig. 1(b) and (d). Challenges such as insufficient information for reconstructing images, shown in Fig. 1(c), or lung movement due to breathing or manipulation of the rigid fibre-bundle during data acquisition, are common. These images with motion artefacts, often referred to as uninformative frames, hinder the clinician's ability to infer useful information from the imaging data [2], [3], [10], and affecting data analysis tasks such as image fusion, classification, and quantitation of different regions of interest. To address these challenges, this paper aims to establish a robust and efficient pipeline for motion artefact compensation in FLIm, specifically tailored for the complexities of real-time pulmonary optical endomicroscopy, ensuring enhanced image quality and reliability for clinical applications.

### A. Related Work

Relevant studies have explored various techniques to address challenges in imaging data generated from OEM systems. Perperidis et al. [10] developed a Gaussian mixture model-based classification for the automatic detection and removal of uninformative frames affected by noise and motion artefacts. This method leverages texture descriptors extracted via the grey-level co-occurrence matrix (GLCM), presenting an effective feature engineering approach for detecting and removing corrupted frames in OEM data. It achieved a sensitivity and specificity of 93%, reducing manual post-analysis effort and improving the robustness of automated workflows.

Perperidis et al. [10] further demonstrated the impact of motion artefacts, where uninformative frames can constitute over 25% of an OEM dataset. Their approach uses principal component analysis to reduce dimensionality and Gaussian models to distinguish informative from corrupted frames. While effective for noise and motion artefacts, this method primarily focuses on frame exclusion rather than addressing spatial displacement, which is critical for real-time imaging improvement in FLIm. Moreover, Sparks et al. [3] introduced an image registration technique to mitigate breathing artefacts, improving the acquisition of intensity images at 8.5 Hz. Their approach utilises maximum Normalised Cross Correlation (NCC) as a similarity metric for image alignment, demonstrating the potential for creating high-quality, alpha-weighted lifetime images. Sparks et al. [3] also implemented time-resolved FLIm with a confocal endomicroscopy, showing that motion artefacts, especially those induced by breathing in small animal models, could be mitigated through frame-by-frame motion tracking using normalized cross-correlation. However, their method excludes low-quality images and focuses primarily on intensity, without providing a comprehensive solution for spatially displaced images or tackling the complex challenges of FLIm registration.

Despite advances in other imaging modalities such as X-ray, MRI, and CT [11], image registration techniques tailored to FLIm remain scarce. Many of these established methods employ NCC as a cost function or similarity metric [11], [12], while other studies used the NCC in object tracking tasks, along with fiducial marker identification and adopted mean squared error minimisation [13], [14]. Yet, these approaches have not fully addressed the intricate structural details or significant spatial displacements typical of real-time pulmonary FLIm imaging, where uncertainties in image matching remain a critical issue. Although attempts have been made to improve computational efficiency and generalisability [15], [16], practical validation in FLIm imaging is still needed.

### B. Contribution

The unique characteristics of FLIm data, such as lower spatial resolution, reduced signal-to-noise ratio, and limited availability, demand sophisticated analysis methods [17], [18]. While machine learning techniques have been employed to enhance FLIm's applicability [1], existing methods, including those by Perperidis et al. [10] and Sparks et al. [3], still face challenges in real-time motion artefact correction.

**Fig. 2.**
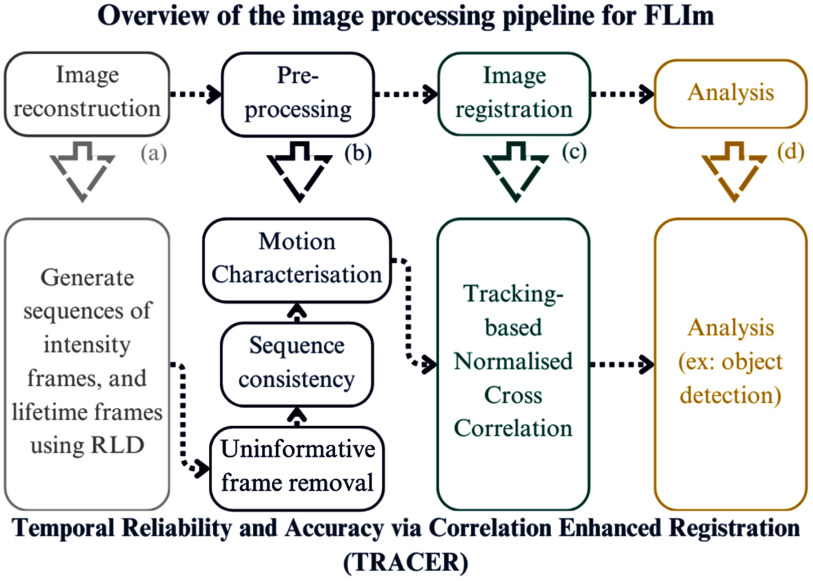
Overview of the TRACER image processing pipeline for FLIm, with emphasis on the proposed TRACER method. (a) Reconstruction of intensity and lifetime frames. (b) Three pre-processing steps ensure consistency and utility of the frames, along with motion model characterisation for the optimal choice of (c), where tracking-based NCC is selected. Finally, (d) FLIm analysis is performed, such as object detection, which is detailed later in the paper.

To address this, we present the TRACER pipeline (Fig. 2), which integrates optimised image registration and motion artefact mitigation techniques, specifically tailored for real-time *in-vivo* pulmonary imaging. A preliminary version of this work has been reported [19]. To the best of our knowledge, no existing pipeline combines these elements with the same level of performance improvement in both processing speed and image quality. These advancements are crucial for maintaining temporal coherence and ensuring reliable data for downstream analysis, particularly in clinical settings where image quality directly influences critical decisions.

Our experiments demonstrate significant enhancements in FLIm image sequence quality and, consequently, improved image fusion. Notably, image registration performance shows up to a 50% improvement across quality of alignment (QA), structural similarity index measure (SSIM), and normalised root mean squared error (NRMSE) metrics for all tested registration methods. Specifically, the novel TRACER registration approach, which integrates Channel and Spatial Reliability Tracker (CSRT) with NCC, outperforms state-of-the-art methods in image registration precision, increasing QA, SSIM, and NRMSE by 5%, 8%, and 3%, respectively, while delivering exceptional computational efficiency—running at approximately an order of a magnitude faster than the next best-performing method across all tested imaging data.

In clinical applications, particularly for object detection tasks, TRACER produces a more reliable fused image, outperforming existing methods, achieving a higher F1 score in positive Neutrophil Activation Probe signal identification within FLIm. These results represent a significant advancement in the utility and quality of real-time FLIm-OEM, with promising implications for improving clinical research outcomes.

### C. Paper Overview

The structure of the paper is as follows: Section II details the data used and the image processing techniques implemented for motion compensation in the TRACER pipeline. Section II-E focuses on the challenges with registering FLIm data. Section III presents the evaluation of pipeline performance through both qualitative and quantitative analyses, and discusses the findings, highlighting key advancements and limitations. Finally, Section V summarises the contributions and suggests directions for future research.

## II. Materials and Methods

Fig. 2 provides an overview of the TRACER pipeline. Specifically, Fig. 2(a) shows the FLIm reconstruction stage, Fig. 2(b) outlines the image sequence pre-processing, Fig. 2(c) illustrates the image registration step, and Fig. 2(d) depicts the application of TRACER-FLIm for downstream image analysis. The pipeline aims to enhance image sequence quality to facilitate image fusion, and support region of interest (ROI) tracking in FLIm data. To understand the algorithmic development, the order of pipeline components, and their impact, we first describe the imaging data as the materials used in this study.

### A. Simulation and Real Data

To evaluate the suitability, efficacy, and limitations of FLIm image processing, six FLIm image sequences and seven simulated sequences of varying complexity were utilised. The raw data were acquired using a custom-built FLIm system [4], capable of capturing fluorescence intensity and lifetime image sequences at up to 8 fps, with each frame having a resolution of 128 × 128 pixels. Lifetime measurements were extracted using the RLD method [17] from the two-time bin intensity data. These lifetime values were then combined with intensity images to generate alpha-weighted lifetime image sequences, enhancing feature interpretability as demonstrated in [3], [20].

The FLIm sequences were selected to represent four common Scenarios in the collected data: (1) homogeneous sequences capturing a single imaged scene, (2) homogeneous sequences with occasional corrupted frames, (3) sequences with corrupted frames and transitions between two distinct scenes, and (4) sequences similar to Scenario 3 but with multiple scene changes caused by natural breathing motion or operator movement of the imaging sensor.

To study these Scenarios, simulated data were generated to analyse challenges such as texture, edges, and recurring structural patterns, which can confound correlation-based algorithms. Using a brick wall texture from the Brodatz database [21] and four high-resolution fluorescence images, seven simulated sequences were created to replicate effects observed in Scenarios 2 and 3, focusing on excluding uninformative frames and detecting scene changes. Sequences 1 to 5 model Scenario 2, featuring small displacements in both $x$ and $y$ directions and occasional uninformative frames. Sequences 6 and 7 replicate Scenario 3, incorporating significant scene changes, corrupted frames, and larger simulated displacements (Supplementary Materials, Fig. 1).

### B. Image Sequence Pre-Processing

The following pre-processing steps constitute an essential component of the novel TRACER pipeline introduced in Section I-B, ensuring the quality and consistency of FLIm image sequences prior to registration. The pre-processing step of the TRACER pipeline consists of two stages: removal of uninformative frames and ensuring sequence consistency. Uninformative frames are removed using the method described in [10], which utilises the feature engineering approach mentioned in Section I-A to detect and discard corrupted frames. Then, to ensure scene consistency across a sequence of $n$ images, where each image is represented as a matrix $\boldsymbol{I}_{[K]}$, $ K = \lbrace 1,\ldots, n\rbrace $, the QA metric introduced in [3] is used. This metric evaluates alignment quality by comparing the image $\boldsymbol{I}_{[K]}$ with the subsequent image $\boldsymbol{I}_{[K+1]}$ in the sequence:

\begin{align*}
QA_{[K+1, K]} = \text{NCC}(\boldsymbol{I}_{[K]}, \boldsymbol{I}_{[K+1]})\,, \tag{1}
\end{align*}

here, QA represents NCC, determined by adapting the NCC function, which can be written as:

\begin{align*}
NCC (\boldsymbol{A},\boldsymbol{B}) = \frac{\sum \nolimits _{(x, y) \in \Omega } \left[(\boldsymbol{A}(x, y) - \mu _{\boldsymbol{A}}) \left(\boldsymbol{B}(x, y) \!-\! \mu _{\boldsymbol{B}}\right)\right]}{\sigma _{\boldsymbol{A}} \cdot \sigma _{\boldsymbol{B}}}\,, \tag{2}
\end{align*}

where $\boldsymbol{A}$ and $\boldsymbol{B}$ are the $\boldsymbol{I}_{[K]}$ and the $\boldsymbol{I}_{[K+1]}$ images, $\boldsymbol{A}(x, y)$ is the scalar image value at pixel coordinates $(x,y)$, $\Omega$ is the set of all pixel coordinates $(x,y)$, and $\mu _{.}$ and $\sigma _{.}$ are the mean and the standard deviation of the respective images. The rate of change (RoC) of the QA metric between consecutive images provides insight into scene consistency. The RoC for images at positions $K$ and $K+1$ in the sequence is defined as:

\begin{align*}
\text{RoC}_{[K]} = \frac{| \text{QA}_{[K+1, K]} - \text{QA}_{[K, K-1]} |}{ \text{QA}_{[K]} } \times 100\,\%\,. \tag{3}
\end{align*}

Scene changes are detected by applying a predefined threshold to the RoC. The sequence is split when the absolute value of the RoC exceeds a threshold value, $\text{thresh}_{RoC}$:

\begin{align*}
|\text{RoC}_{[K]}| \geq \text{thresh}_{RoC}\,\%\,. \tag{4}
\end{align*}

**Fig. 3.**
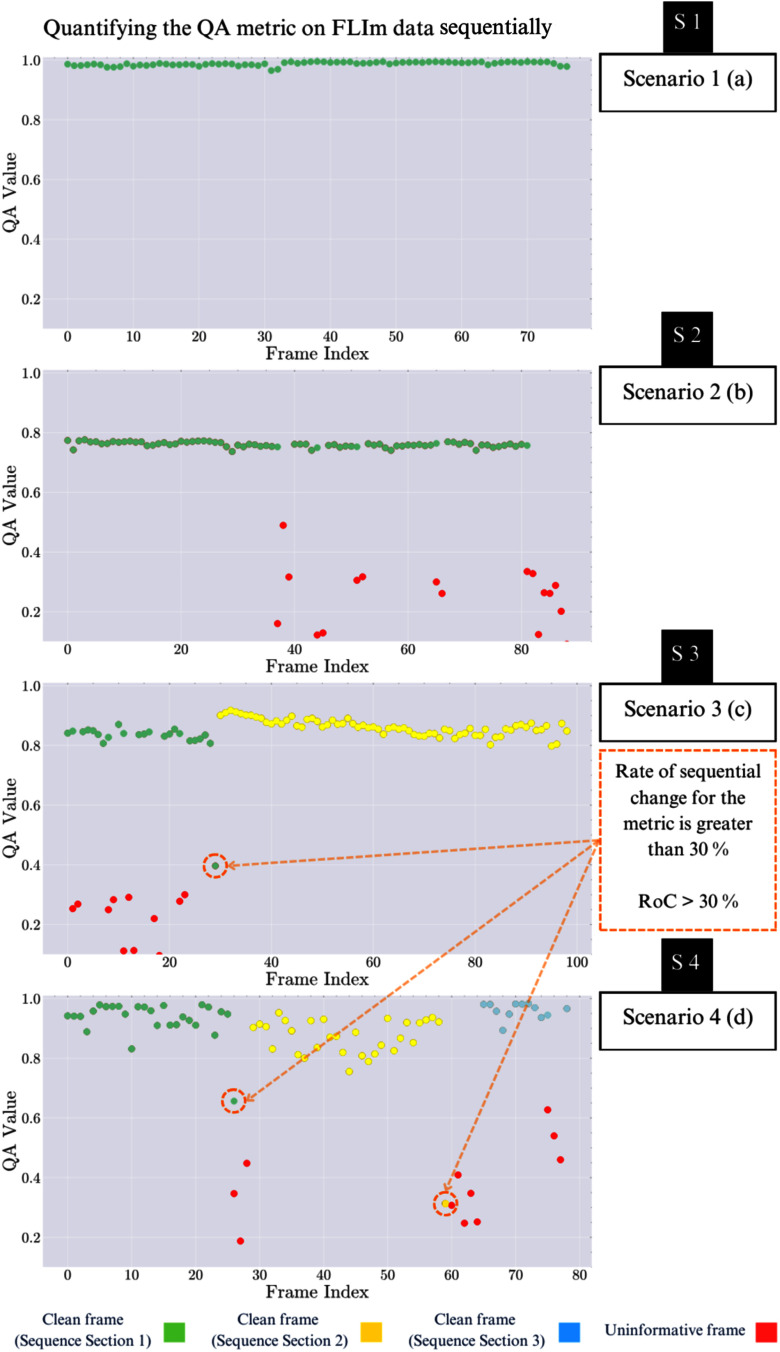
Sequential quantification of the QA metric for the FLIm image sequences from the four Scenarios described in Section II-A. (a) Represents a homogeneous sequence as in Scenario 1, where each point corresponds to the QA between consecutive frames. In Scenario 2, with occasional uninformative frames, the plot appears as in (b). Red points indicate uninformative frames, which, when removed, allow for clearer detection of significant changes (RoC $\geq$ 30 %) in Scenarios 3 and 4, shown in (c) and (d), respectively. This highlights transitions between distinct scenes.

Upon empirically analysing the RoC across the sequences described in Section II-A, it was found that a threshold value of $\text{thresh}_{RoC} \approx 30\,\%$, or greater, typically corresponds to a transition between different scenes, such as those caused by operator movement of the imaging sensor. By implementing this consistency check, we ensure that the analysed sequence depicts a stable scene, enhancing the reliability of subsequent image processing steps. See Fig. 3 for details on the quantification of RoC across the different sequence Scenarios.

### C. Image-to-Image Characterisation

The next step of the TRACER pipeline is the characterisation of the motion between the frames in the sequence. Based on observations from the available FLIm data, rigid and translation-based image registration was assumed adequate. To further validate and quantify this, the optical flow method presented in [22] provides a robust two-frame motion estimation algorithm that characterises image-to-image pixel displacement, which is crucial for understanding the motion model before applying image registration. Details of estimating the image-to-image motion can be found in the Supplementary Materials.

### D. Image Registration

Generally, the task of image registration involves a process aimed at achieving geometric alignment between two images: a reference image $\boldsymbol{I}_{[K]}(\boldsymbol{\chi })\,\lbrace \boldsymbol{\chi }=(x,y)\rbrace$, and a moving image ${\boldsymbol{I}_{[K+1]}(\boldsymbol{u})\,\lbrace \boldsymbol{u}=(u_,v)\rbrace }$, where $\boldsymbol{\chi }$ and $\boldsymbol{u}$ represent the spatial coordinates associated with the reference and moving image, respectively. The alignment is typically realised through a transformation function $\boldsymbol{T}$ that maps the coordinates $\boldsymbol{u}$ of $\boldsymbol{I}_{[K+1]}$ to $\boldsymbol{\chi }$ in $\boldsymbol{I}_{[K]}$.

Image registration techniques can be broadly categorised into rigid and non-rigid methods. Rigid registration assumes that a set of rigid body transformations such as translation and rotation can describe the transformation between the two images. This is often suitable for applications where the imaged objects are rigid and maintain their shape. Conversely, non-rigid registration allows for more complex, deformable transformations, accounting for variations in shape and structure across the image [12]. Both types of image registration are usually applied using two different approaches:

#### 1) Feature-Based Registration

Feature-based registration involves extracting distinct elements (e.g., regions, lines, points) from an image pair and matching them for alignment [23]. This approach can be problematic when such features are scarce, making it difficult to establish correspondences. Additionally, transformation function estimation may suffer if features are absent or mismatched [24]. These limitations are significant in medical imaging [12], [24], including FLIm, where distinct features can be difficult to identify due to the dynamic nature of the imaging sequences and the requirement for features to remain constant throughout the sequence [24].

#### 2) Similarity-Based Registration

This approach omits the feature detection step and focuses on matching selected regions or even entire images between the reference and moving images [23]. Typically, similarity-based methods are framed as an optimisation problem [12], where the aim is to find a transformation function that maximises the similarity between the reference and moving images, often employing metrics such as the normalised cross-correlation (NCC), (2).

At that point, (2) is used to include the spatial transformation $\boldsymbol{T}$ as $\text{NCC}(\boldsymbol{I}_{[K]}(\boldsymbol{\chi }), \boldsymbol{T}(\boldsymbol{I}_{[K+1]}))$, where, $\boldsymbol{T}$ is the transformation function mapping $\boldsymbol{\chi }$ to $u$, aligning $\boldsymbol{I}_{[K+1]}$ with $\boldsymbol{I}_{[K]}$.

$\boldsymbol{I}_{reg}$ is defined as the composition, represented by $\circ$, of the optimal transformation function $\boldsymbol{T^{*}}$ with the moving image $\boldsymbol{I}_{[K+1]}$, $\boldsymbol{I}_{reg} = \boldsymbol{I}_{[K+1]} \circ \boldsymbol{T^{*}}$. Typically, $\boldsymbol{T^{*}}$ can be found by iteratively optimising a similarity function such as the NCC [12], [25] and can be written as:

\begin{align*}
\boldsymbol{T^{*}} = \arg \max _{\boldsymbol{T}} \, \text{NCC}(\boldsymbol{I}_{[K]}, \boldsymbol{T}(\boldsymbol{I}_{[K+1]})) \tag{5}
\end{align*}

This can be solved using an optimisation algorithm, such as the Powell method [12], which is a widely used optimisation method that does not require the computation of gradients, which is advantageous when dealing with complex or non-differentiable objective functions, such as those often encountered in image registration. We refer to the image registration represented by (5) as NCC-General registration. Similarly, the Enhanced Correlation Coefficient (ECC) is a well-established, state-of-the-art, metric that can also be used. It is calculated as the correlation coefficient between the input and reference images, enhanced by a weighting function that prioritises pixels closer to the mean intensity, thereby increasing robustness to lighting changes [26]. Moreover, Mattes Mutual Information (MMI) is another approach that analyses the statistical dependence between the two images by comparing their joint pixel intensity histogram to their individual histograms and calculating a score based on the entropy of these histograms [27]. When well-aligned, both MMI and ECC scores are maximised, indicating greater similarity and shared information between the images. Furthermore, the study in [28] used optical flow (OF) to determine the optimal transformation for image alignment. The algorithm here approximates the pixel displacement from the reference to the moving image, which is then used to compute a velocity field describing the apparent pixel motion. This field warps the moving image to align it with the reference image.

However, as outlined in [11], [24], estimating the type of motion between images can enable direct image registration methods, eliminating the need for iterative solutions to determine the transformation function. That is, $\boldsymbol{T}$ in (5) can be restricted to linear translation parameters when considering a translation motion model. We can also directly use the NCC offset between consecutive image pairs, performing a NCC-Translate image registration. Similarly, the ECC can also be restricted to translation only.

Nonetheless, our empirical analysis revealed that the unique properties of FLIm, combined with significant displacements, present notable challenges for image registration. This is particularly true for optimisation processes, even when restricted to translation. Moreover, methods involving the NCC become problematic when dealing with multiple peaks in the NCC space, complicating accurate offset computation.

### E. The Proposed Image Registration Approach

This section represent the core novelty of the TRACER pipeline described in Section I-B. It investigates the challenges of registering the image sequences described in Section II-A after pre-processing using the two steps mentioned in Section II-B, and characterise the appropriate motion model.

#### 1) Multiple Peaks in Normalised Cross-Correlation

Considering the direct method to achieve image registration and to understand the issue of multiple peaks in the NCC space, we first define the NCC 2D map $\mathbf {C}_{map}$. Utilising the Fourier domain variant of the NCC function provides a computationally efficient method for calculating and analysing the NCC $2D$ arrays, as described in [29]. Using this approach, (2) can be rewritten to compute $\mathbf {C}_{map}$ as follows:

\begin{align*}
\mathbf {C}_{map}(\boldsymbol{I}_{K}, \boldsymbol{I}_{K+1}) = \frac{\mathcal {F}^{-1}\left[\mathcal {F}(\boldsymbol{I}_{[K+1]}^{H})^{*} \times \mathcal {F}(\boldsymbol{I}_{[K]})\right]}{\sigma _{\boldsymbol{I}_{[K]}} \cdot \sigma _{\boldsymbol{I}_{[K+1]}}}\,, \tag{6}
\end{align*}

where $\sigma _{\boldsymbol{I}_{[K]}}$ and $\sigma _{\boldsymbol{I}_{[K+1]}}$ are the standard deviation of the reference and moving images, respectively:

\begin{align*}
\begin{split} \sigma _{\boldsymbol{I}_{[K]}} &= \sqrt{\sum \nolimits_{(x,y)\in \Omega }\Bigl (\boldsymbol{I}_{[K]}(x,y)-\mu _{\boldsymbol{I}_{[K]}}\Bigr)^{2}}, \\
 \sigma _{\boldsymbol{I}_{[K+1]}} &= \sqrt{\sum \nolimits_{(x,y)\in \Omega }\Bigl (\boldsymbol{I}_{[K+1]}(x,y)-\mu _{\boldsymbol{I}_{[K+1]}}\Bigr)^{2}} \end{split} \tag{7}
\end{align*}

$\mathcal {F}$ represents the Fast Fourier Transform, and $\mathcal {F}^{-1}$ is its inverse. $\boldsymbol{I}_{[K+1]}^{H}$ denotes the horizontally flipped version of $\boldsymbol{I}_{[K+1]}$, and $*$ indicates the complex conjugate, following the definition of cross-correlation rather than convolution [30].

**Fig. 4.**
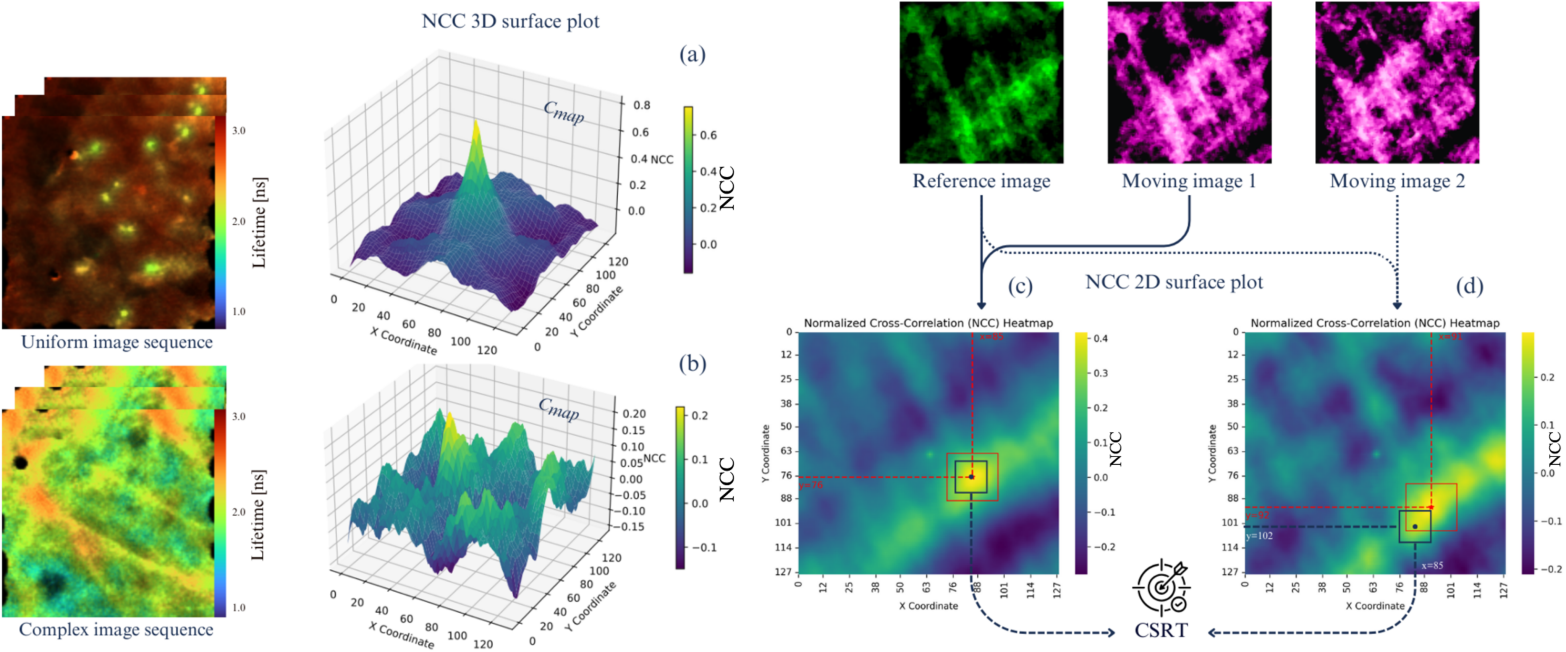
This figure illustrates the effect of image complexity on NCC and the resulting $\mathbf {C}_{map}$, along with the role of CSRT in addressing issues caused by multiple peaks. (a) shows a $\mathbf {C}_{map}$ with a single dominant peak, due to the low complexity in FLIm image Sequence 1. In contrast, (b) and (c) display $\mathbf {C}_{map}$s from FLIm image Sequences 2 and 3, where increased complexity results in multiple peaks. (c) also shows the 2D $\mathbf {C}_{map}$ used for tracking. When a single dominant peak exists, the identified peak (red square) coincides with the peak tracked by CSRT (black square). However, (d) illustrates the difficulty of identifying the correct peak in a $\mathbf {C}_{map}$ with multiple peaks, highlighting how CSRT ensures accurate tracking and localisation of the correct peak (black square) for precise computation of translation parameters.

Examination of the $\mathbf {C}_{map}$ produced by the FLIm sequences revealed a noisy NCC landscape with multiple peaks, as seen in Fig. 4(b), primarily caused by edges and repetitive structures dominating the image correlation. Moreover, the controlled experiments using simulation image sequences also produced $\mathbf {C}_{map}$s with multiple peaks when computing NCC between reference and moving images in sequences 1, 5, and 7. These factors contribute to the challenge encountered during image registration and highlight the influence of image complexities, such as repetitive patterns and large displacements.

#### 2) Channel and Spatial Reliability Peak Tracking Registration

Fig. 4 demonstrates the impact of image complexity on the NCC space and highlights the necessity of a robust tracking mechanism to accurately identify the correct correlation peak. In simpler cases, such as Sequence 1 (Fig. 4(a)), the $\mathbf {C}_{map}$ contains a single dominant peak, allowing straightforward identification of the correct translation between the reference and moving images. However, for more complex image sequences, as seen in Fig. 4(b) and (c), multiple peaks emerge in the $\mathbf {C}_{map}$, often due to repetitive structural elements or significant displacements, which complicates the accurate localisation of the peak representing the true transformation. Therefore, without a robust tracking mechanism, the identified peak (red square) may lead to errors in image registration.

To mitigate this, the CSRT [31] is integrated with the NCC for the image registration task. The CSRT algorithm enhances the traditional Discriminative Correlation Filter (DCF) [32] by incorporating spatial and channel reliability mechanisms, enabling robust tracking even in complex Scenarios with multiple similar features. Here, the CSRT is used to maintain consistent tracking of the correct peak (black square), even in the presence of multiple peaks, as shown in Fig. 4(d). This approach ensures the reliable computation of translation parameters, enhancing the robustness of the image registration process, especially in challenging conditions where the NCC landscape is noisy and contains several competing peaks.

In our approach, the dominant peak in the NCC map $\mathbf {C}_{\text{map}}$ is tracked across frames to maintain alignment accuracy. Initially, $\mathbf {C}_{\text{map}}[0]$ is used to determine the location of the true dominant peak $(i_{0}, j_{0})$. Subsequently, the NCC is computed between a selected reference frame $\mathbf {I}_{[K]}$ (typically the first frame in the sequence) and each moving image $\mathbf {I}_{[K+1]}$ in a sequence of $n$ images, generating a series of NCC maps $\mathbf {C}_{\text{map}}[k]$, where $k \in \lbrace 1, 2,\ldots, n-1\rbrace$. The objective is to track the peak representing the correct alignment across these maps, despite the presence of multiple peaks caused by repetitive patterns or large displacements.

The CSRT approach learns a set of channel-specific DCFs to localise the dominant peak across frames. Let $c \in \lbrace 1, \ldots, C\rbrace$ index the feature channels extracted from the correlation map $\mathbf {C}_{\text{map}}$, where $C$ is the total number of channels. At the initial frame $\mathbf {C}_{\text{map}}[0]$, feature channels $\lbrace \mathbf {\xi }_{c}\rbrace _{c=1}^{C}$ are extracted, while at subsequent frames $\mathbf {C}_{\text{map}}[k]$, $\lbrace \mathbf {x}_{c}\rbrace _{c=1}^{C}$ are extracted.

The CSRT formulates this tracking problem by learning a DCF for each channel, minimising the following objective:

\begin{align*}
\underset{\lbrace \mathbf {DCF}_{c}\rbrace }{\arg \min } \left\Vert \mathbf {S} \odot \!\left(\! \boldsymbol{\rho } - \sum _{c=1}^{C} \mathbf {\xi }_{c} \ast \mathbf {DCF}_{c} \!\right) \right\Vert ^{2} \!+\! \lambda \sum _{c=1}^{C} R_{c}\left\Vert \mathbf {DCF}_{c} \right\Vert ^{2}, \tag{8}
\end{align*}

where:

• $\mathbf {\xi }_{c}$: the $c$-th feature channel extracted from $\mathbf {C}_{\text{map}}[0]$,
• $\mathbf {DCF}_{c}$: the filter for the $c$-th channel,
• $\boldsymbol{\rho }$: the desired response, typically a Gaussian centred at the target location,
• $\mathbf {S}$: the spatial reliability map, indicating the reliability of each region,
• $R_{c}$: the reliability score for the $c$-th channel,
• $\lambda$: a regularisation parameter,
• $\odot$: element-wise multiplication,
• $\ast$: convolution.

The spatial reliability map $\mathbf {S}$ focuses on the region around the dominant peak in $\mathbf {C}_{\text{map}}[0]$, and for subsequent frames $\mathbf {C}_{\text{map}}[k]$ is defined as:

\begin{align*}
\mathbf {S}(i, j) = \exp \left(-\frac{(i - i_{0})^{2} + (j - j_{0})^{2}}{2 \sigma _{s}^{2}} \right), \tag{9}
\end{align*}

where $(i_{0}, j_{0})$ is the location of the dominant peak in $\mathbf {C}_{\text{map}}[0]$, and $\sigma _{s}$ controls the spatial decay.

The channel reliability $R_{c}$ determines how each channel contributes to the final response:

\begin{align*}
R_{c} = \exp \left(-\frac{\Vert \mathbf {d}_{c}\Vert ^{2}}{2 \sigma _{c}^{2}} \right), \tag{10}
\end{align*}

where $\mathbf {d}_{c}$ represents the difference between the feature descriptors of the target in the current and previous $\mathbf {C}_{\text{map}}$ for the $c$-th channel, and $\sigma _{c}$ controls how quickly the channel reliability decays. Solving (8) yields filters $\lbrace \mathbf {DCF}_{c}\rbrace$ that emphasise reliable channels and spatial regions, aiding the tracker in following the dominant peak across frames, even when multiple peaks exist.

Once the tracker is updated, the dominant peak for $\mathbf {C}_{\text{map}}[k]$ is obtained by:

\begin{align*}
(i_{\text{peak}}, j_{\text{peak}}) = \arg \max _{(i, j)} \left(\mathbf {S} \odot \sum _{c=1}^{C} \mathbf {x}_{c} \ast \mathbf {DCF}_{c} \right)(i, j). \tag{11}
\end{align*}

Here, $\mathbf {x}_{c}$ denotes the $c$-th feature channel extracted from $\mathbf {C}_{\text{map}}[k]$. Finally, the transformation $\boldsymbol{T}$ is determined using the offset $(\Delta i_{k}, \Delta j_{k}) = (i_{\text{peak}} - i_{0}, j_{\text{peak}} - j_{0})$, and the registered image is obtained as:

\begin{align*}
\mathbf {I}_{\text{reg}[k]} = \mathbf {I}_{[k+1]} \circ \boldsymbol{T}_{(\Delta i_{k}, \Delta j_{k})}. \tag{12}
\end{align*}

Our evaluation suggests that registration errors predominantly arise from the misidentification of the true optimal NCC peak, caused by the presence of competing secondary peaks with similar prominence, e.g. Fig. 4(b). Similarly, using the ECC method on complex image sequences confirmed convergence errors. Upon examining the 3D NCC map, multiple peaks were identified, complicating the registration process. By tracking the dominant peak using CSRT, a robust and efficient alignment is achieved without the need for iterative optimisation of similarity metrics, which can be particularly challenging for FLIm data due to image complexities.

## III. Results

Recent studies, such as [11], [25], have highlighted the need to tailor these methods to the specific data and applications to ensure suitability. The TRACER pipeline was designed with this consideration in mind: by carefully characterising the motion model between images before the registration stage, the most appropriate approach can be selected. Therefore, offering a comprehensive yet straightforward solution for processing FLIm images to fuse temporal sequences, enabling the tracking and quantification of objects within the image scenes.

Our findings suggest that rigid transformation is effective for aligning image pairs within the TRACER pipeline. To demonstrate this, the proposed registration approach in TRACER is compared against the methods described in Section II-D: NCC-Translate, NCC-General, ECC, MMI, and OF. An ablation study was conducted, evaluating registration performance with and without each step of the TRACER pipeline to assess the methods and the impact of the pre-processing steps.

The evaluation was based on three key metrics: QA, SSIM [33], and NRMSE, and was conducted on all the image sequences described in Section II-A.

### A. Quantitative Analysis

The TRACER pipeline comprises two primary pre-processing steps, as detailed in Section II-B. Step 1 involves the removal of uninformative frames—–those that do not contribute to downstream analysis—–while Step 2 ensures sequence consistency by detecting and addressing scene changes throughout the sequence.

**Fig. 5.**
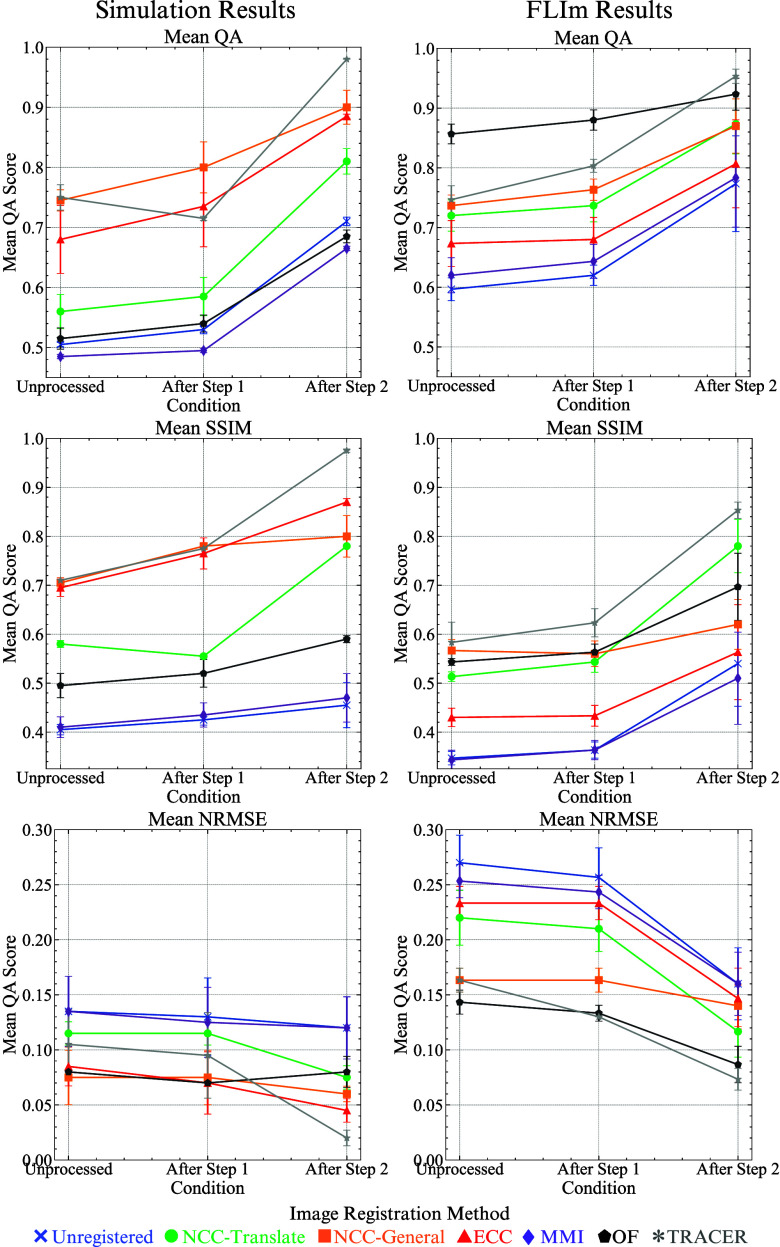
This figure illustrates the effect of the two primary pre-processing steps (1 and 2) described in Section II-B on the QA, SSIM, and NRMSE metrics. The error bars on the left represent the progressive improvements in the average metric values for the simulation sequences following each step, while those on the right display the corresponding results for the FLIm datasets. Detailed statistical analyses, including means and standard deviations demonstrating the significance and consistency of these improvements, are provided in Supplementary Materials Section III-C and Tables II– VI.

As shown in Fig. 5, for simulation sequences, before Step 1 (Unprocessed), no registration method achieves optimal performance without Step 1. However, after applying step 1 and removing uninformative frames, QA improves by 4.3%, SSIM by 5.6%, and NRMSE by 2.6% on average across all registration methods. Though, TRACER trails NCC-General and ECC due to tracking errors introduced by new scenes, underscoring the necessity of Step 2.

For FLIm data, OF generally performs best, particularly in sequences containing multiple scenes. This is because, unlike simulated sequences—where scene differences are pronounced and localised registration errors in MMI and OF lead to feature deformations negatively impacting metrics—FLIm images from different scenes exhibit greater similarity in appearance. Consequently, deformative methods like OF are more adept at addressing these subtle variations (Supplementary Materials, Table II and Table III).

Notably, after applying Step 2, registration performance improves significantly across all methods, with increases of 15.6% in QA, 30.5% in SSIM, and 19.4% in NRMSE (Supplementary Materials, Table IV and Table V). Overall, the TRACER pipeline enhances registration performance by up to 50% on average across all registration methods and metrics. The proposed registration approach consistently outperforms state-of-the-art methods, achieving average improvements of 5% in QA, 8% in SSIM, and 3% in NRMSE when both Steps are applied (Further ablation details are available in Supplementary Materials, Sections III-A and III-B).

### B. Computational Runtime

While both OF and MMI methods successfully achieved temporal registration, OF proved to be computationally intensive and lacked precision in cases where the deformative nature of the technique was not needed. Moreover, the MMI suffered from sensitivity to initial conditions and susceptibility to local optima due to its reliance on gradient descent for maximising mutual information between image pairs [34].

**Fig. 6.**
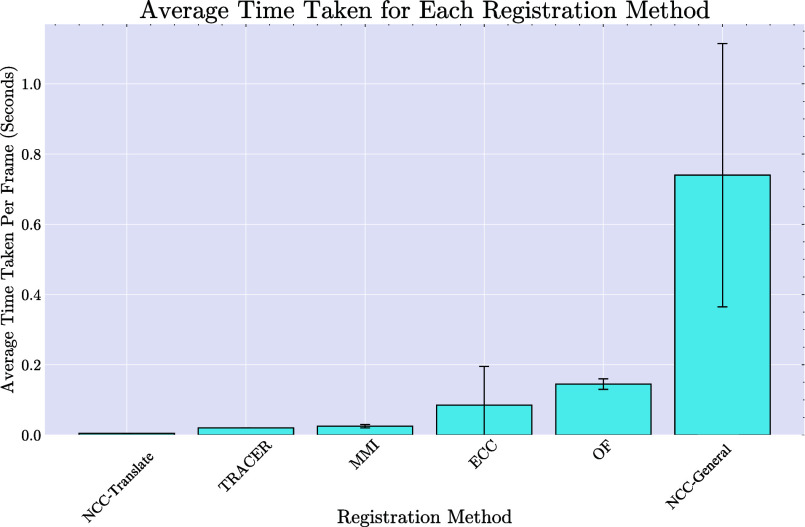
Comparison of the average time required to register a pair of images using the six registration methods benchmarked in this study, applied across all simulation and FLIm imaging datasets. Error bars represent the standard deviation across the experiments.

Focusing the motion model on translational movements, primarily driven by natural respiratory motions, NCC-General and ECC demonstrated comparable or, in some cases, more accurate results. By using the NCC-Translate method to compute registration parameters, the computational complexity commonly associated with iterative optimisation in image registration can be mitigated, as seen with NCC-General. The TRACER registration approach, on the other hand, maintains high accuracy and offers a computationally efficient solution, achieving about an order of magnitude faster performance than the next most accurate registration method. The computational runtime for registering each pair of images was measured using an Intel Core i7-6700 CPU with 8 GB of RAM, see Fig. 6 for details.

Balancing runtime efficiency and accuracy is particularly important in real-time medical imaging, not just to improve speed but also to lower hardware costs, making devices more accessible. Additionally, reducing computational demands contributes to minimising environmental impact, a growing priority for future technologies.

### C. Application Example to Segmentation and Detection

**Fig. 7.**
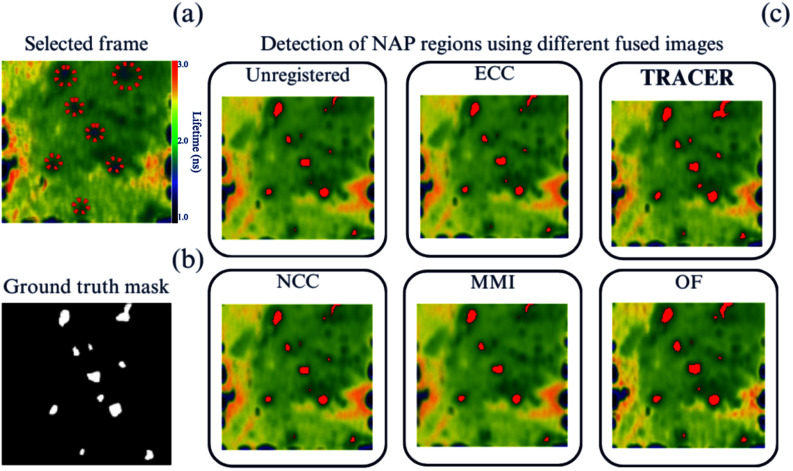
Evaluation of NAP object detection, contrasting the efficacy of the proposed TRACER with outcomes from alternative image registration techniques. (a) Presents a sample image from the sequence with dotted red circles highlighting the targeted NAP ROIs. (b) Displays the ground truth binary mask. (c) Depicts the superimposed masks on the fused images, highlighting the detected regions in red.

To further verify the efficacy of the image registration task, this segmentation and detection experiment aimed to evaluate the performance of the detection of Neutrophil Activation Probe (NAP) signals [9] on the resulting fused images. A clinical expert provided a ground truth binary mask, shown in Fig. 7(b), highlighting areas of positive NAP signal throughout the image sequence for benchmarking purposes.

The detection methodology entails the application of a predefined threshold to segregate positive NAP signals within the FLIm imaging, as delineated by criteria established in prior studies [7], [9]. Positive regions manifest as distinct objects against the FLIm image contrast, exemplified in Fig. 7(a). This method resulted in the generation of binary masks from fused images derived through the various registration techniques, as shown in Fig. 7(c). The efficacy of each registration method was quantitatively assessed, and results are shown in Table I using precision, recall, and F1-score (also known as the Dice coefficient or DICE) measures, widely used metrics for assessing the accuracy of object detection algorithms [35]. Details are provided in Supplementary Materials.

**TABLE I:** Comparison of NAP Detection in Fused Images Using Various Registration Techniques, Including the Proposed TRACER Method

Method	Evaluation Metric		
	Precision $\uparrow$	Recall $\uparrow$	F1 score $\uparrow$
Unregistered	0.91 $\pm$ 0.07	0.63 $\pm$ 0.06	0.75 $\pm$ 0.08
NCC-Translate	0.92 $\pm$ 0.08	0.61 $\pm$ 0.05	0.72 $\pm$ 0.07
NCC-General	0.92 $\pm$ 0.06	0.61 $\pm$ 0.06	0.73 $\pm$ 0.08
ECC	0.92 $\pm$ 0.07	0.60 $\pm$ 0.09	0.73 $\pm$ 0.04
MMI	0.89 $\pm$ 0.08	0.50 $\pm$ 0.05	0.64 $\pm$ 0.07
OF	0.79 $\pm$ 0.09	0.68 $\pm$ 0.08	0.73 $\pm$ 0.04
TRACER	**0.93 $\pm$ 0.04**	**0.88 $\pm$ 0.04**	**0.90 $\pm$ 0.08**

Reported Values are the Mean Performance Scores, With $\pm$ Representing the Standard Deviation. Further Statistical Analyses Can Be Found in Supplementary Materials.

Table I revealed that TRACER stands out with increases in precision by up to 4 % and recall by up to 27 %. Additionally, the higher F1 score indicates a better balance between identifying relevant ROI and minimising erroneous detection, with up to a 19 % improvement seen with the image example, fused after applying the TRACER registration method. This demonstrates the enhanced accuracy in identifying positive NAP signals within the FLIm imaging data example, validating the effectiveness of the proposed registration approach.

## IV. Discussion

While the TRACER pipeline robustly processes FLIm data, several enhancements are still needed. Currently, it employs intensity-weighted-lifetime data, which, although interpretable, limits analysis to a single modality. A key improvement is the integration of learning-based image registration tailored for FLIm. Deep learning has proven effective in multi-modal registration: for example, Wang et al. [20] proposed a co-registration approach aligning FLIm with histology data using an optimisation-based regression network. Similarly, remote sensing techniques, such as the multi-scale Convolutional Neural Networks (CNNs) feature descriptor by Yang et al. [36], underscore the need for robust multi-temporal feature representations, while Li et al. [37] demonstrated enhanced tracking via deep adaptive networks incorporating respiratory motion compensation. Adapting these frameworks could improve FLIm motion compensation in dynamic conditions, though the limited availability of annotated datasets remains a challenge—one that might be mitigated through unsupervised or weakly supervised methods, domain adaptation, or synthetic augmentation.

Future work could integrate adaptive frameworks that automatically classify motion complexity and select optimal registration strategies. By incorporating such machine learning techniques, TRACER could evolve into a more flexible and intelligent system, ultimately enhancing its clinical applicability.

Another limitation concerns the handling of more complex motions. While the current rigid transformation approach has been successful for the tested sequences, it does not fully address cases involving significant rotation, scaling, or affine transformations, partly due to the limited availability of FLIm datasets. Expanding the dataset and incorporating non-rigid registration techniques will be crucial to managing more dynamic motion patterns. Furthermore, the characterisation of image-to-image motion has been instrumental in this study, as it helps select appropriate registration techniques based on motion characteristics. Future work could extend this by developing an adaptive framework where motion characterisation guides the choice of registration method. For instance, low-displacement sequences could use simpler translation-based approaches, while more complex motions may require deformable registration techniques.

Lastly, the current image fusion strategy, which relies on averaging, is effective for noise reduction but may fail to capture critical details when objects of interest are small, dynamic, and modelled as point spread functions [38]. This could result in the loss of vital information. Future efforts will focus on refining fusion techniques that maintain noise reduction while preserving essential details in FLIm analysis. This will accurately represent static and dynamic features, improving the pipeline's clinical relevance.

## V. Conclusion

This paper introduces an enhanced image processing pipeline, TRACER, designed to mitigate motion artefacts in real-time Optical Endomicroscopy FLIm, addressing a critical challenge in current research. The TRACER pipeline offers an efficient and accurate solution for FLIm image processing by incorporating pre-processing steps to remove uninformative frames, leveraging dense optical flow for motion characterisation, and employing Channel and Spatial Reliability Tracker for a tracking-based Normalised Cross Correlation registration approach. This pipeline significantly improves registration and image fusion, enabling precise alignment even in sequences with large displacements and multiple scenes.

Empirical results, as demonstrated in Fig. 5, show that TRACER achieves 20% to 30% improvement in QA, SSIM, and NRMSE metrics, with Step 1 and Step 2 contributing substantially to this image registration performance gain. Furthermore, the TRACER registration approach consistently surpasses state-of-the-art image registration methods, delivering average improvements of 5% in QA, 8% in SSIM, and 3% in NRMSE. In addition to its accuracy, TRACER significantly reduces computational time, operating approximately an order of magnitude faster than the next best-performing method. The combination of these factors positions TRACER as a valuable tool for real-time FLIm image analysis.

The pipeline's robustness and real-time applicability are demonstrated by its strong performance on both simulated and real FLIm imaging sequences. Its efficient handling of complex motion patterns underscores its potential for broader clinical and research applications. Future work will extend the dataset and incorporate non-rigid registration, particularly in the alveolar region, to manage more intricate motion. Further refinements in image fusion and optimisation of motion models and parameter selection will also enhance the pipeline's performance.

In summary, TRACER makes a significant contribution to FLIm-specific image processing by resolving the unique challenges of FLIm registration. It substantially improves the quality and reliability of real-time FLIm imaging through enhanced temporal alignment and the removal of uninformative frames—both critical factors for accurate registration. This leads to tangible quantitative benefits and directly improves clinical interpretation, enabling more robust downstream analyses such as precise NAP signal detection. Consequently, TRACER represents a valuable advance in FLIm image processing with direct implications for improved clinical diagnostics.

## Supplementary Materials

Supplementary Materials

## Author Contributions

T. H. and J. R. H. conceived and designed the methodology presented in this study. K. D. facilitated access to the materials and experimental infrastructure required for data acquisition. All authors contributed to the critical revision of the manuscript. All authors have read and agreed to the published version of the manuscript.

## Conflict of Interest

The authors declare that they have no conflicts of interest relevant to the content of this article.

## References

[1] D. Gouzou , “Applications of machine learning in time-domain fluorescence lifetime imaging: A review,” Methods Appl. Fluorescence, vol. 12, no. 022001, 2024, doi: 10.1088/2050-6120/ad12f7. PMC10851337 38055998

[2] A. Perperidis , “Image computing for fibre-bundle endomicroscopy: A review,” Med. Image Anal., vol. 62, 2020, Art. no. 101620, doi: 10.1016/j.media.2019.101620. PMC7611433 32279053

[3] H. Sparks , “Heterogeneity in tumor chromatin-doxorubicin binding revealed by in vivo fluorescence lifetime imaging confocal endomicroscopy,” Nature Commun., vol. 9, 2018, Art. no. 2662. 10.1038/s41467-018-04820-6 PMC6037736 29985394

[4] E. Williams , “High speed spectral fluorescence lifetime imaging for life science applications,” Proc. SPIE, vol. 10889, 2019, Art. no. 108890P, doi: 10.1117/12.2509466.

[5] A. Taimori, D. Humphries, G. Williams, K. Dhaliwal, N. Finlayson, and J. Hopgood, “Fast and robust single-exponential decay recovery from noisy fluorescence lifetime imaging,” IEEE Trans. Biomed. Eng., vol. 69, no. 12, pp. 3703–3716, Dec. 2022, doi: 10.1109/TBME.2022.3176224. 35609109

[6] A. Taimori , “A novel fit-flexible fluorescence soft imager: Tri-sensing of intensity, fall-time, and life profile,” IEEE Trans. Biomed. Eng., vol. 71, no. 6, pp. 1864–1878, Jun. 2024, doi: 10.1109/TBME.2024.3354856. 38300773

[7] D. C. Humphries , “Specific in situ immuno-imaging of pulmonary-resident memory lymphocytes in human lungs,” Front. Immunol., vol. 14, 2023, Art. no. 1100161, doi: 10.3389/fimmu.2023.1100161. PMC9951616 36845117

[8] J. R. Lakowicz, Principles of Fluorescence Spectroscopy, J. R. Lakowicz, Ed. Boston, MA, USA: Springer, 2006, doi: 10.1007/978-0-387-46312-4.

[9] T. H. Craven , “Activated neutrophil fluorescent imaging technique for human lungs,” Sci. Reports, vol. 11, 2021, Art. no. 976, doi: 10.1038/s41598-020-80083-w. PMC7806726 33441792

[10] A. Perperidis , “Automated detection of uninformative frames in pulmonary optical endomicroscopy,” IEEE Trans. Biomed. Eng., vol. 64, no. 1, pp. 87–98, Jan. 2017, doi: 10.1109/tbme.2016.2538084. 26978410

[11] S. Abbasi , “Medical image registration using unsupervised deep neural network: A scoping literature review,” Biomed. Signal Process. Control, vol. 73, 2022, Art. no. 103444, doi: 10.1016/j.bspc.2021.103444.

[12] G. Song , “A review on medical image registration as an optimization problem,” Curr. Med. Imag. Rev., vol. 13, no. 3, pp. 274–283, 2017, doi: 10.2174/1573405612666160920123955. PMC5543570 28845149

[13] Z. Liu, C. Huang, and J. Luo, “A systematic investigation of lateral estimation using various interpolation approaches in conventional ultrasound imaging,” IEEE Trans. Ultrason., Ferroelectr., Freq. Control, vol. 64, no. 8, pp. 1149–1160, Aug. 2017, doi: 10.1109/TUFFC.2017.2705186. 28534769

[14] P. Sebastian and Y. V. Voon, “Tracking using normalized cross correlation and color space,” in Proc. 2007 Int. Conf. Intell. Adv. Syst., Nov. 2007, pp. 770–774, doi: 10.1109/ICIAS.2007.4658490.

[15] A. Masullo , “On dealing with multiple correlation peaks in PIV,” Experiments Fluids, vol. 59, 2018, Art. no. 89, doi: 10.1007/s00348-018-2542-z.

[16] D. Caulfield , “Track validation using gradient-based normalised cross-correlation,” in Proc. Brit. Mach. Vis. Conf. 2011. Brit. Mach. Vis. Assoc., 2011, pp. 70.1–70.11, doi: 10.5244/C.25.70.

[17] X. Liu , “Fast fluorescence lifetime imaging techniques: A review on challenge and development,” J. Innov. Opt. Health Sci., vol. 12, no. 05, 2019, Art. no. 1930003, doi: 10.1142/S1793545819300039.

[18] S. Guo , “FLIM data analysis based on laguerre polynomial decomposition and machine-learning,” J. Biomed. Opt., vol. 26, no. 2, 2021, Art. no. 022909, doi: 10.1117/1.JBO.26.2.022909. PMC7790506 33415850

[19] T. Haloubi, S. A. Thomas, C. Hines, K. Dhaliwal, and J. R. Hopgood, “Navigating noise and texture: Motion compensation methodology for fluorescence lifetime imaging in pulmonary research,” in Proc. IEEE 46th Annu. Int. Conf. Eng. Med. Biol. Soc., Jul. 2024, pp. 1–5, doi: 10.1109/embc53108.2024.10781956. 40039579

[20] Q. Wang , “Deep learning-assisted co-registration of full-spectral autofluorescence lifetime microscopic images with h&e-stained histology images,” Commun. Biol., vol. 5, 2022, Art. no. 1119, doi: 10.1038/s42003-022-04090-5. PMC9586936 36271298

[21] U. of Southern , “Broadtz brick wall texture dataset,” 2025, Accessed: Mar. 16, 2025. [Online]. Available: https://sipi.usc.edu/database/database.php?volume=textures

[22] G. Farnebäck, “Two-frame motion estimation based on polynomial expansion,” in Image Analysis, J. Bigun and T. Gustavsson, Eds. Berlin, Germany: Springer, 2003, pp. 363–370, doi: 10.1007/3-540-45103-X_50.

[23] B. Zitová, “Image registration methods: A survey,” Image Vis. Comput., vol. 21, no. 11, pp. 977–1000, 2003. [Online]. Available: https://www.sciencedirect.com/science/article/pii/S0262885603001379

[24] A. I. Abidi , Deformable registration techniques for thoracic CT images: An insight into medical image registration. Singapore: Springer, 2020, doi: 10.1007/978-981-10-5837-0.

[25] Y. Fu , “Deep learning in medical image registration: A review,” Phys. Med. Biol., vol. 65, 2020, Art. no. 20TR01, doi: 10.1088/1361-6560/ab843e. PMC7759388 32217829

[26] G. D. Evangelidis and E. Z. Psarakis, “Parametric image alignment using enhanced correlation coefficient maximization,” IEEE Trans. Pattern Anal. Mach. Intell., vol. 30, no. 10, pp. 1858–1865, Oct. 2008, doi: 10.1109/TPAMI.2008.113. 18703836

[27] D. Mattes, D. R. Haynor, H. Vesselle, T. K. Lewellen, and W. Eubank, “PET-CT image registration in the chest using free-form deformations,” IEEE Trans. Med. Imag., vol. 22, no. 1, pp. 120–128, Jan. 2003, doi: 10.1109/TMI.2003.809072. 12703765

[28] Y. Douini , “Solving sub-pixel image registration problems using phase correlation and Lucas-Kanade optical flow method,” in Proc. 2017 Intell. Syst. Comput. Vis., Apr. 2017, pp. 1–5, doi: 10.1109/ISACV.2017.8054948.

[29] J. TANG , “Fast template matching algorithm,” J. Comput. Appl., vol. 30, pp. 1559–1561, 2010. [Online]. Available: https://api.semanticscholar.org/CorpusID

[30] J.-C. Yoo , “Fast normalized cross-correlation,” Circuits, Syst. Signal Process., vol. 28, pp. 819–843, 2009, doi: 10.1007/s00034-009-9130-7.

[31] A. Lukežič , “Discriminative correlation filter tracker with channel and spatial reliability,” Int. J. Comput. Vis., vol. 126, pp. 671–688, 2018, doi: 10.1007/s11263-017-1061-3.

[32] J. F. Henriques, R. Caseiro, P. Martins, and J. Batista, “High-speed tracking with kernelized correlation filters,” IEEE Trans. Pattern Anal. Mach. Intell., vol. 37, no. 3, pp. 583–596, Mar. 2015, doi: 10.1109/CVPR.2018.00512. 26353263

[33] Z. Wang, A. C. Bovik, H. R. Sheikh, and E. P. Simoncelli, “Image quality assessment: From error visibility to structural similarity,” IEEE Trans. Image Process., vol. 13, no. 4, pp. 600–612, Apr. 2004, doi: 10.1109/TIP.2003.819861. 15376593

[34] A. Sotiras, C. Davatzikos, and N. Paragios, “Deformable medical image registration: A survey,” IEEE Trans. Med. Imag., vol. 32, no. 7, pp. 1153–1190, Jul. 2013.. 10.1109/TMI.2013.2265603 PMC3745275 23739795

[35] A. Géron, Hands-on Machine Learning With Scikit-Learn, Keras, and TensorFlow. Sebastopol, CA, USA: O'Reilly Media, Inc., 2022, doi: 10.5555/3378999.

[36] Z. Yang, T. Dan, and Y. Yang, “Multi-temporal remote sensing image registration using deep convolutional features,” IEEE Access, vol. 6, pp. 38544–38555, 2018, doi: 10.1109/ACCESS.2018.2853100.

[37] M.-D. Li , “ADMNet: Adaptive-weighting dual mapping for online tracking with respiratory motion estimation in contrast-enhanced ultrasound,” IEEE Trans. Image Process., vol. 33, pp. 58–68, 2024, doi: 10.1109/TIP.2023.3333195. 37988213

[38] M. Demirel, B. Mills, E. Gaughan, K. Dhaliwal, and J. R. Hopgood, “Bayesian statistical analysis for bacterial detection in pulmonary endomicroscopic fluorescence lifetime imaging,” IEEE Trans. Image Process., vol. 33, pp. 1241–1256, 2024, doi: 10.1109/tip.2024.3361217. 38324436

