# Supplementary material for: Motion Compensation in Pulmonary Fluorescence Lifetime Imaging: An Image Processing Pipeline for Artefact Reduction and Clinical Precision
Source: IEEE Open J Eng Med Biol. 2025 Apr 8;6:432–41. doi: 10.1109/OJEMB.2025.3558620 (PMC12251131; doi:10.1109/OJEMB.2025.3558620)
Supplement: Supplementary Materials [file supp1-3558620.pdf]

Supplementary Materials

Motion Compensation in Pulmonary Fluorescence Lifetime Imaging: An Image Processing Pipeline for Artefact Reduction and Clinical Precision

Tarek Haloubi *Member, IEEE*, Spencer Angus Thomas *Senior Member, IEEE*, Catherine Hines, Kevin Dhaliwal, James R. Hopgood *Senior Member, IEEE*

I. Supplemental Material on Simulation and Real Data

The figure (Fig. 1) provides a visual summary of the simulated and FLIm sequences analysed in this study, illustrating varying degrees of sequence variability. Sequence variability is categorised into four Scenarios (S1 to S4), with colour-coded squares marking the level of variation. S1 represents the least variable sequences, containing only clean frames (green squares). S2 introduces moderate variability with occasional uninformative frames (red squares). S3 displays higher variability, characterised by uninformative frames and transitions between two distinct scenes (yellow squares for the second scene). S4, the most variable, includes multiple scene changes (blue squares for the third scene) caused by operator-induced fibre bundle movement.

II. Supplemental Material on Image-to-image Characterisation

This algorithm in [1] approximates the neighbourhood of each frame using quadratic polynomials through a polynomial expansion transform, allowing efficient characterisation of image motion by observing how a polynomial representing a small neighbourhood around a pixel transform under different motion models.

The displacement field between images is estimated from the coefficients of these polynomial expansions. Specifically, the method involves:

1) Approximating each image neighbourhood with quadratic polynomials using the polynomial expansion transform.
2) Estimating the displacement field by analysing changes in polynomial coefficients due to motion.
3) Refining this estimate through iterative and multi-scale strategies to achieve robust motion characterisation.

The mathematical formulation of the polynomial expansion for a pixel's neighbourhood is expressed as:

$$\begin{aligned} f(x,y) \approx & f(x_0,y_0) + [x-x_0, y-y_0]\begin{bmatrix} f_x \\ f_y \end{bmatrix} \\ & + \frac{1}{2}[x-x_0,y-y_0]\begin{bmatrix} f_{xx} & f_{xy} \\ f_{xy} & f_{yy}\end{bmatrix}[x-x_0,y-y_0]^T , \end{aligned} \tag{1}$$

where $f(x,y)$ represents the intensity function of the image $\boldsymbol{I}_{[K]}$, (x_0,y_0) is the centre of the neighbourhood, and f_x, f_y, f_{xx}, f_{xy}, and f_{yy} are the polynomial coefficients corresponding to the first- and second-order derivatives of the image intensity function. These coefficients capture the local structure of the image and variations caused by motion. From the polynomial expansion in (1), motion-induced changes in the image intensity between consecutive frames can be modelled by estimating the displacement vector $[x-x_0, y-y_0]$ for each pixel. By calculating the derivatives f_x and f_y, the intensity gradient along the x and y directions can be determined, forming the basis for computing the displacement field. The motion vectors, derived from this displacement field, quantify the local movement of pixels and can be collectively represented as the vector field $\boldsymbol{V}$, where each vector $\boldsymbol{v}_i = (v_{x_i}, v_{y_i})$ encodes the pixel displacement in the x and y directions.

To facilitate the interpretation of the vector field $\boldsymbol{V}$, polar coordinate colour maps are employed [2]. Fig. 2(a) illustrates these maps, where the direction of motion, represented by the angle θ_i of each vector $\boldsymbol{v}_i$, is mapped to hue on the colour wheel. Simultaneously, the magnitude $|\boldsymbol{v}_i|$ of each vector, indicating the speed of motion, is represented by intensity or saturation.

In the case of pure translation, the vectors in $\boldsymbol{V}$ point in the same direction, reflecting a consistent motion across the frame. However, variations in real-world scenarios, such as noise, occlusions, or non-rigid movements, introduce deviations. To quantify the coherence of the motion model, the angular variance of the motion vectors, σ_θ^2, is calculated as:

$$\sigma_\theta^2 = \frac{1}{Z}\sum_{z=1}^{Z}(\theta_z - \bar{\theta})^2 , \tag{2}$$

where θ_z is the angle (in degrees) of the zth motion vector relative to a reference axis, $\bar{\theta}$ is the mean angle of all vectors in $\boldsymbol{V}$, and Z is the total number of motion vectors.

This equation directly relates to the vector field $\boldsymbol{V}$ by capturing the angular distribution of all vectors in the field. A lower σ_θ^2 indicates that the motion vectors are closely aligned, suggesting that the motion is well approximated by a translational model. Conversely, a higher σ_θ^2 signifies more diverse motion directions, indicative of non-translational or complex motion patterns.

Table I
SUMMARY OF MOTION VECTORS' ANGULAR VARIANCE ACROSS DIFFERENT IMAGING SEQUENCES.

Image sequences	Mean Motion vectors σ_θ^2
Simulation sequences	for translation* mostly $< 1.2°$
FLIm sequence 1	$0.18° \pm 0.13°$
FLIm sequence 2	$0.36° \pm 0.06°$
FLIm sequence 3	$0.29° \pm 0.05°$
FLIm sequence 4	$0.62° \pm 0.09°$
FLIm sequence 5	$0.41° \pm 0.13°$
FLIm sequence 6	$0.97° \pm 0.12°$

* See Fig. 2(b) for a visual representation of σ_θ^2 for simulated sequences.

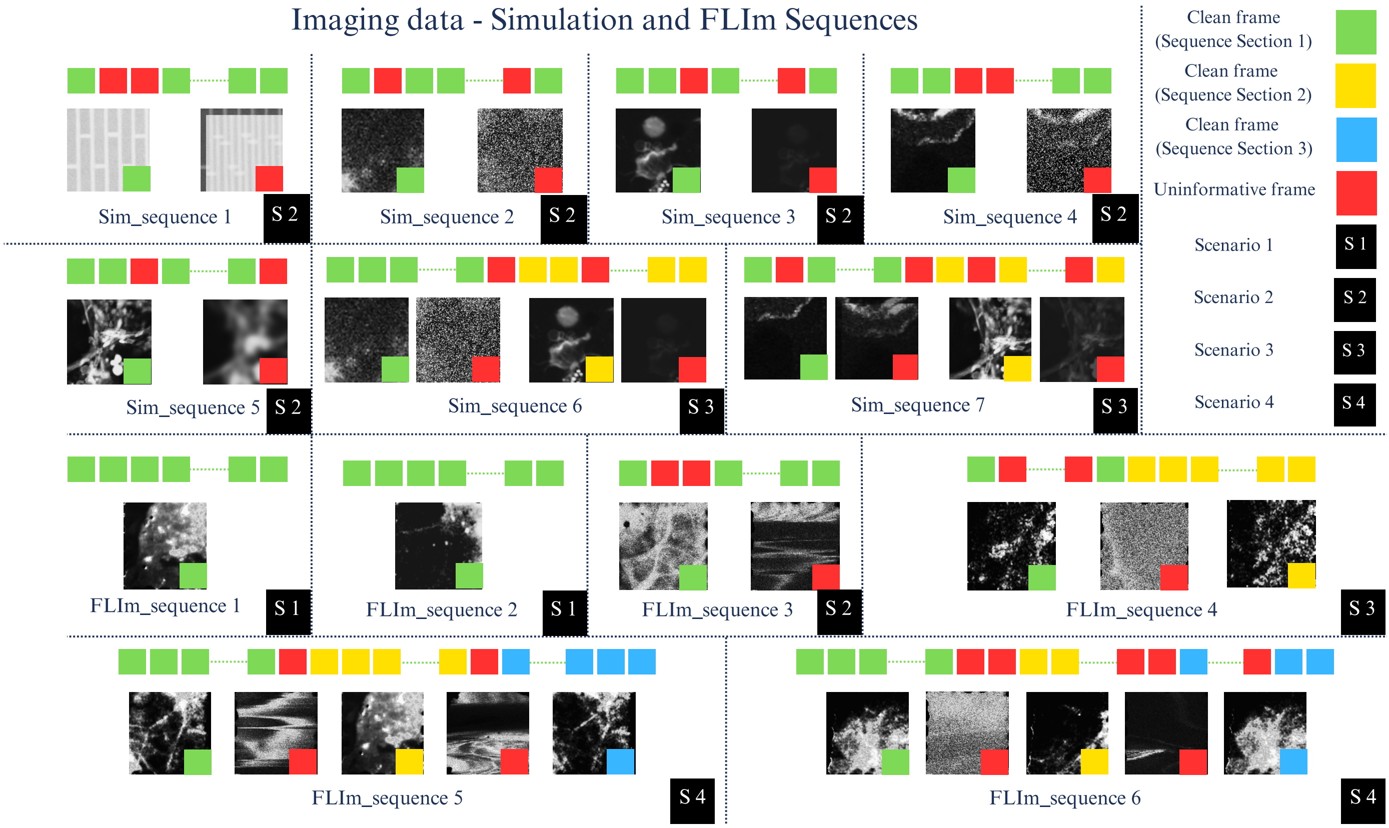

Figure 1. Overview of the simulated and FLIm sequences used in this study. The colour-coded squares above the image examples indicate sequence variability. Scenario 1 (S1) sequences are the least variable, consisting of clean frames (green squares). Scenario 2 (S2) sequences show moderate variability, with occasional uninformative frames (red squares). Scenario 3 (S3) sequences exhibit increased variability, featuring both uninformative frames and transitions between two distinct scenes (yellow squares for the second scene). Scenario 4 (S4) sequences are the most variable, involving multiple scene changes (blue squares for the third scene) due to operator movement of the fibre bundle.

The utility of σ_θ^2 as a metric is evident in Fig. 2(b), where angular variance values are shown for five simulated sequences subjected to different motion models, including translation, rotation, scaling, and affine transformations. As demonstrated, sequences with translational motion exhibit the lowest σ_θ^2, reflecting their uniform directional characteristics.

Moreover, the metric is applied to assess motion consistency in the six FLIm image sequences analysed in this study. Table I shows that the lower σ_θ^2 values align with the observations made during data acquisition, confirming the predominance of a translational motion model in these sequences.

III. Supplementary Results

The following subsections and tables provide detailed metrics and descriptions of the ablation for simulation and FLIm sequences before and after applying the 2 primary steps described in Section II-B in the main paper. Step 1 involves removing uninformative frames—those that are not useful for downstream analysis tasks, and step 2 ensures sequence consistency by detecting changes in the scene along the sequence.

A. Step 1 Ablation

Table II presents the quantitative image registration results for the quality of alignment (QA), structural similarity index measure (SSIM), and normalised root mean squared error (NRMSE) metrics on the simulated sequences before and after applying Step 1. As shown by the Table II, Temporal Reliability and Accuracy via Correlation Enhanced Registration (TRACER) marginally outperforms other methods, with improvements of 6.5 % in QA, 7.6 % in SSIM, and 1.5 % in NRMSE. However, none of the registration methods perform as well without applying Step 1. After removing uninformative frames, QA improves by 4.3 %, SSIM by 5.6 %, and NRMSE by 27.6 % on average across all registration methods. notably, TRACER is the only method capable of perfectly compensating for the small translational shifts observed across the initial five temporal sequences. In contrast, the Enhanced Correlation Coefficient (ECC) method succeeds in achieving this only within the context of simulation sequence 3.

The errors observed in the Normalised Cross Correlation (NCC)-Translate, NCC, and ECC methods are primarily due to incorrect localisation of the optimal solution. Additionally, localised registration errors in Mattes Mutual Information (MMI) and optical flow (OF) result in feature deformations within the images, negatively affecting metric evaluations.

When considering FLIm data, Table III shows that for image sequences 1 and 2, Step 1 has no effect on registration performance. This is expected, as these sequences fall under Scenario 1, where no uninformative frames are present. For

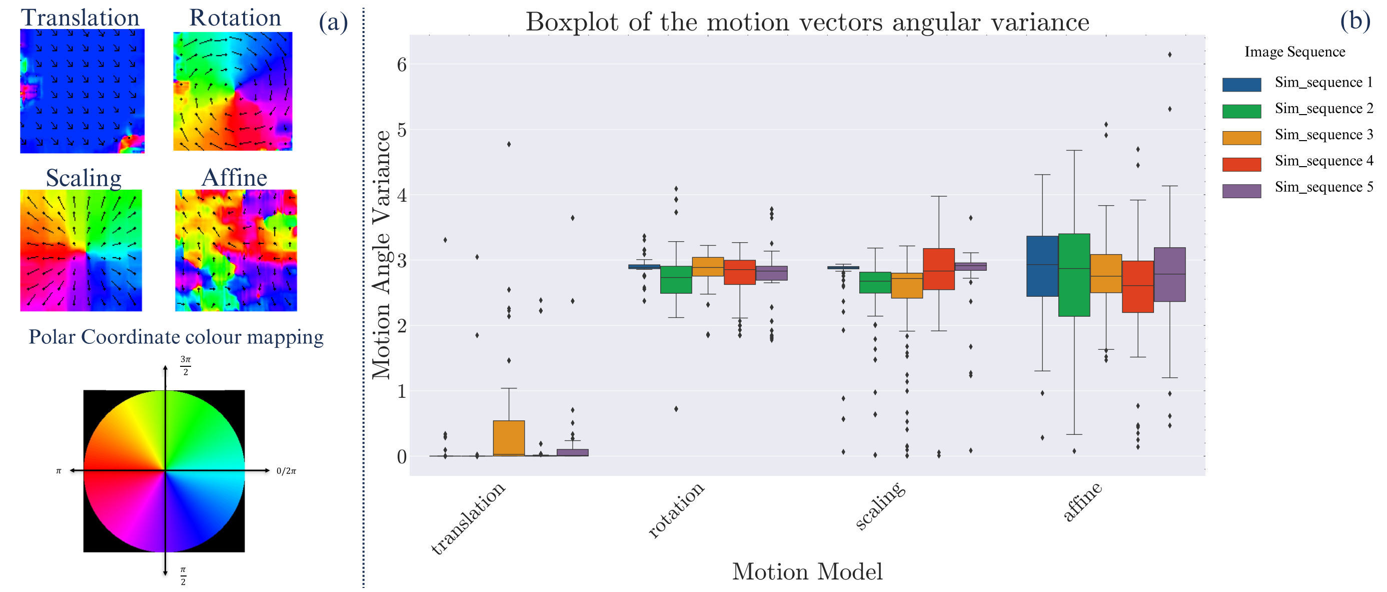

Figure 2. Evaluation of image-to-image motion characterisation using optical flow on simulated sequences. (a) Polar coordinate colour maps representing motion direction and magnitude: The angle of each motion vector is colour-coded according to the circular hue spectrum (0° mapped to red, 90° green, 180° cyan, and 270° purple), illustrating directionality, while magnitude (speed) of motion vectors is represented by colour saturation—more vivid colours indicate higher speed, whereas less saturated (paler) colours indicate lower speeds. (b) Angular variance of motion vectors across five simulated sequences, where translational motion shows the lowest angular variance, confirming consistent directional characteristics.

the remaining sequences (3 to 6), OF generally performs the best, especially in sequences with multiple scenes. This is because, unlike the simulated sequences where scenes are entirely different, the FLIm images from different scenes are more similar in appearance. In such cases, deformative methods like OF are better suited to handle those subtle changes.

However, this observation holds before applying step 2 and primarily for QA. When looking at the SSIM metric, which is more sensitive to structural deformations [3], [4], OF falls behind other methods. The deformative nature of OF can introduce distortions that negatively impact the structural similarity between images, as reflected in the SSIM scores.

B. Step 2 Ablation

Table IV compares the performance of different image registration methods across the QA, SSIM, and NRMSE metrics before and after applying Step 2 for sequences that require this step, such as simulation sequences 6 and 7. As shown in Table IV, registration performance struggles with these sequences compared to those benefiting only from Step 1 (simulation sequences 1 to 5). However, after applying Step 2, registration performance improves significantly across all methods—by 15.6 % in QA, 30.5 % in SSIM, and 19.4 % in NRMSE. TRACER outperforms other methods, leading by 3.0 % in QA, 10.5 % in SSIM, and 2.5 % in NRMSE over the next best registration approach.

For the FLIm data, Table V presents the results for sequences 4 to 6 before and after applying Step 2. The application of Step 2 results in notable improvements—16.6 % in QA, 31.2 % in SSIM, and 35.4 % in NRMSE on average across all registration methods. Here, TRACER registration maintains its lead, outperforming the next best method by 1.6 % in QA, 7.3 % in SSIM, and 1.3 % in NRMSE.

C. Statistical Assessment of Registration Performance

Table VI summarises the statistical analysis of the mean improvements for QA, SSIM, and NRMSE across FLIm sequences 4, 5, and 6, before and after implementing Steps 1 and 2 of the TRACER pipeline. The analysis was performed with paired t-tests using the SciPy stats library [5] to determine whether the observed performance gains were statistically significant at the $p < 0.05$ level.

From Table VI, most registration methods exhibit clear and statistically significant improvements in all three metrics (QA, SSIM, NRMSE) once the TRACER pipeline's pre-processing is applied. For instance, the Unregistered case improves from a mean QA of 0.513 to 0.781, with $p = 0.012$, indicating that the simple act of removing uninformative frames and ensuring scene consistency yields a notable benefit before any actual image registration is performed. Similar significant gains are observed for NCC-General ($p = 0.000$), ECC ($p = 0.015$), MMI ($p = 0.012$), and OF ($p = 0.005$), confirming that the pipeline contributes substantially to improving alignment quality and structural fidelity.

A notable exception is NCC-Direct, whose increases (e.g. from QA=0.701 to 0.792) did not meet the threshold for statistical significance ($p = 0.411$). Closer inspection suggests that although some numerical gains are observed, they do not consistently outweigh the within-sequence variation when assessed by a paired t-test. This highlights that while the pipeline can facilitate better registration for most methods, its overall impact may vary when a method's core function has limited robustness against multiple peaks or intensity variations in certain FLIm frames.

Among all methods, our proposed TRACER approach shows particularly strong statistical significance ($p = 0.001$), with QA rising from 0.668 to 0.940 and SSIM from 0.506 to 0.850.

This confirms that TRACER's dedicated peak-tracking strategy, coupled with the prior removal of uninformative frames and splitting of inconsistent scene segments, yields the largest and most consistent benefits for rigid registration in FLIm data. Consequently, these results illustrate that carefully designed pre-processing steps can substantially aid most registration algorithms, and the final TRACER registration stage provides an additional, statistically validated improvement beyond those of standard methods.

D. Qualitative Example

Fig. 3 illustrates the fusing of a pair of reference and moving images from FLIm image sequence 3, shown in green and magenta, respectively. A perfect alignment results in a grayscale image, indicating precise alignment, whereas misalignment is characterised by green and magenta discrepancies, as demonstrated in Fig. 3(a). Additionally, Fig. 3 presents registration results fused with the reference image, scored by the QA. The results were obtained using the proposed TRACER registration approach alongside the other methods mentioned earlier. Notably, the TRACER method exhibits superior registration accuracy in Fig. 3(f), outperforming the other methods based on the quality of the fused image.

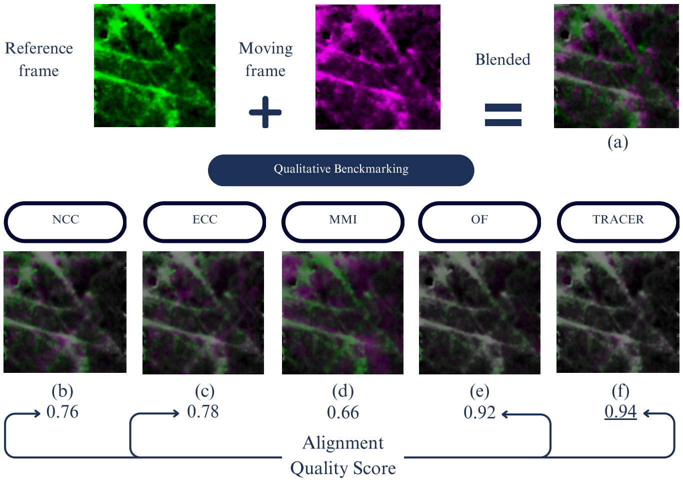

Figure 3. Evaluation of registration outcomes. The reference and moving images are represented in green and magenta, respectively. Perfect alignment results in a grayscale image, as opposed to the misalignment depicted in (a). Sub-figures (b) to (f) showcase the most successful alignments from the benchmarking experiment: (b) and (c) display results using NCC (same result as NCC-Translate) and ECC, respectively; (d) and (e) present outcomes via MMI and OF, respectively; and (f) illustrates the alignment achieved with TRACER.

E. Application Example to Segmentation and Detection

Precision represents the ratio of the true positive predictions to the total positive predictions and is defined as:

$$\text{Precision} = \frac{TP}{TP + FP}, \quad \text{Recall} = \frac{TP}{TP + FN} \tag{3}$$

where TP denotes the true positives, FP represents the false positives, and FN denotes the false negatives. Recall is the ratio of the true positive predictions to the total number of relevant object predictions.

The true positives (TP), false positives (FP), and false negatives (FN) are derived from comparing the ground truth binary mask with the binary mask generated by the detection method. This comparison is executed as follows:

- TP are pixels where both the predicted mask and the ground truth mask agree on a positive Neutrophil Activation Probe (NAP) signal, represented as pixels with a value of 1 in both masks.
- FP are pixels identified as positive signals in the predicted mask but are not present in the ground truth mask.
- FN represents the missed positive signals, where the predicted mask shows no signal despite the presence of a signal in the ground truth mask.

Table VII summarises the statistical comparison of the proposed TRACER method against other registration techniques, specifically focusing on Precision, Recall, and the F1-score metrics. Precision alone, while informative, primarily reflects false positives (FP) and does not account for false negatives (FN). Thus, Recall, which accounts for FN, and the F1-Score, the harmonic mean of Precision and Recall, provide a more comprehensive assessment of detection performance:

$$F1 = 2 \times \frac{\text{Precision} \times \text{Recall}}{\text{Precision} + \text{Recall}} \tag{4}$$

This metric effectively summarises the overall accuracy, balancing both false positives and false negatives. Statistical analysis (Table VII) demonstrates that the proposed TRACER method significantly improves detection performance ($p < 0.05$) compared to NCC-Translate, MMI, and OF. However, no statistically significant differences were found between TRACER and the Unregistered, NCC-General, or ECC methods for Precision.

Notably, TRACER achieves consistently higher Recall and F1-Score, underscoring its superior ability to minimise false negatives and improve overall detection robustness. These findings strongly support the adoption of the proposed registration method for clinical applications requiring reliable and accurate image fusion.

References

[1] G. Farnebäck, "Two-frame motion estimation based on polynomial expansion," in *Image Analysis*, J. Bigun *et al.*, Eds. Berlin, Heidelberg: Springer Berlin Heidelberg, 2003, pp. 363–370. [Online]. Available: https://doi.org/10.1007/3-540-45103-X_50
[2] Y. Adato *et al.*, "A polar representation of motion and implications for optical flow," in *CVPR 2011*, June 2011, pp. 1145–1152. [Online]. Available: https://doi.org/10.1109/CVPR.2011.5995419
[3] Z. Wang *et al.*, "Image quality assessment: From error visibility to structural similarity," *IEEE Transactions on Image Processing*, vol. 13, pp. 600–612, 4 2004. [Online]. Available: https://doi.org/10.1109/TIP.2003.819861
[4] J. C. Mier *et al.*, "Deep perceptual image quality assessment for compression," in *2021 IEEE International Conference on Image Processing (ICIP)*, Sep. 2021, pp. 1484–1488.
[5] SciPy, "ttest_rel — scipy v1.15.2 manual." [Online]. Available: https://docs.scipy.org/doc/scipy/reference/generated/scipy.stats.ttest_rel.html

Table II
IMPACT OF STEP 1 ON REGISTRATION METHODS AND TRACER, USING SIMULATION IMAGE SEQUENCES.

Data	Method	Without Step 1			With Step 1			Metric Δ		
		QA ↑	SSIM ↑	NRMSE ↓	QA ↑	SSIM ↑	NRMSE ↓	QA	SSIM	NRMSE
Sim-seq 1	Unregistered	0.79 ± 0.22	0.21 ± 0.22	0.16 ± 0.21	0.86 ± 0.07	0.21 ± 0.23	0.10 ± 0.06	0.06	0.00	0.06
	NCC-Direct	0.55 ± 0.38	0.75 ± 0.24	0.21 ± 0.21	0.52 ± 0.37	0.82 ± 0.16	0.19 ± 0.17	-0.03	0.07	0.02
	NCC-General	0.68 ± 0.16	0.41 ± 0.28	0.13 ± 0.17	0.86 ± 0.08	0.36 ± 0.26	0.09 ± 0.06	0.06	-0.05	0.04
	ECC	**0.80** ± 0.19	0.74 ± 0.25	0.14 ± 0.23	0.86 ± 0.12	0.83 ± 0.15	0.04 ± 0.04	0.06	0.09	0.10
	MMI	0.60 ± 0.18	0.24 ± 0.24	0.21 ± 0.12	0.64 ± 0.12	0.22 ± 0.22	0.21 ± 0.13	0.04	-0.02	0.00
	OF	0.77 ± 0.16	0.68 ± 0.23	0.13 ± 0.21	0.82 ± 0.11	0.75 ± 0.17	0.05 ± 0.04	0.05	0.07	0.08
	TRACER	**0.80** ± 0.19	**0.91** ± 0.19	**0.10** ± 0.18	**1.00** ± 0.00	**1.00** ± 0.00	**0.00** ± 0.00	0.20	0.09	0.10
Sim-seq 2	Unregistered	0.73 ± 0.19	0.43 ± 0.13	0.07 ± 0.08	0.76 ± 0.15	0.46 ± 0.09	0.06 ± 0.03	0.03	0.03	0.01
	NCC-Direct	0.61 ± 0.35	0.72 ± 0.26	0.12 ± 0.08	0.55 ± 0.34	0.78 ± 0.20	0.08 ± 0.07	-0.06	0.06	0.01
	NCC-General	0.67 ± 0.12	0.71 ± 0.19	0.11 ± 0.04	0.92 ± 0.05	0.71 ± 0.20	0.03 ± 0.02	0.03	0.00	0.01
	ECC	**0.79** ± 0.25	**0.75** ± 0.28	**0.05** ± 0.07	0.84 ± 0.22	0.82 ± 0.24	0.03 ± 0.03	0.05	0.07	0.02
	MMI	0.64 ± 0.17	0.46 ± 0.11	0.12 ± 0.07	0.68 ± 0.14	0.46 ± 0.08	0.06 ± 0.04	0.04	0.00	0.01
	OF	0.69 ± 0.20	0.52 ± 0.21	0.06 ± 0.08	0.71 ± 0.17	0.56 ± 0.19	0.05 ± 0.03	0.02	0.04	0.01
	TRACER	0.75 ± 0.17	0.73 ± 0.20	0.06 ± 0.07	**1.00** ± 0.00	**1.00** ± 0.00	**0.00** ± 0.00	0.25	0.27	0.06
Sim-seq 3	Unregistered	0.84 ± 0.18	0.61 ± 0.17	0.09 ± 0.09	0.88 ± 0.12	0.65 ± 0.07	0.08 ± 0.04	0.04	0.04	0.01
	NCC-Direct	0.73 ± 0.23	0.74 ± 0.23	0.06 ± 0.07	0.74 ± 0.21	0.79 ± 0.19	0.06 ± 0.05	0.01	0.05	0.00
	NCC-General	0.77 ± 0.14	0.73 ± 0.17	0.06 ± 0.06	0.95 ± 0.05	0.75 ± 0.13	0.06 ± 0.04	0.05	0.02	0.00
	ECC	0.81 ± 0.16	0.79 ± 0.23	**0.04** ± 0.07	**1.00** ± 0.00	**1.00** ± 0.00	**0.00** ± 0.01	0.19	0.21	0.04
	MMI	0.82 ± 0.16	0.63 ± 0.17	0.09 ± 0.07	0.86 ± 0.11	0.66 ± 0.11	0.08 ± 0.05	0.04	0.03	0.01
	OF	0.88 ± 0.15	0.67 ± 0.20	0.07 ± 0.06	0.92 ± 0.05	0.72 ± 0.13	0.06 ± 0.04	0.04	0.05	0.01
	TRACER	**0.92** ± 0.17	**0.89** ± 0.21	0.05 ± 0.08	**1.00** ± 0.00	**1.00** ± 0.00	**0.00** ± 0.00	0.08	0.11	0.05
Sim-seq 4	Unregistered	0.64 ± 0.21	0.50 ± 0.15	0.08 ± 0.09	0.67 ± 0.18	0.53 ± 0.10	0.06 ± 0.04	0.03	0.03	0.02
	NCC-Direct	0.80 ± 0.20	0.73 ± 0.27	0.05 ± 0.07	0.83 ± 0.16	0.79 ± 0.20	0.04 ± 0.03	0.03	0.06	0.01
	NCC-General	0.88 ± 0.15	0.85 ± 0.18	0.06 ± 0.05	0.97 ± 0.05	0.89 ± 0.14	0.02 ± 0.02	0.04	0.04	0.01
	ECC	0.72 ± 0.26	0.71 ± 0.27	0.06 ± 0.07	0.77 ± 0.24	0.78 ± 0.23	0.04 ± 0.04	0.05	0.07	0.02
	MMI	0.64 ± 0.21	0.47 ± 0.12	0.09 ± 0.10	0.66 ± 0.19	0.49 ± 0.06	0.07 ± 0.03	0.02	0.02	0.02
	OF	0.70 ± 0.18	0.57 ± 0.20	0.06 ± 0.06	0.73 ± 0.15	0.62 ± 0.16	0.05 ± 0.03	0.03	0.05	0.01
	TRACER	**0.90** ± 0.22	**0.90** ± 0.21	**0.04** ± 0.08	**1.00** ± 0.00	**1.00** ± 0.00	**0.00** ± 0.00	0.10	0.10	0.04
Sim-seq 5	Unregistered	0.86 ± 0.11	0.25 ± 0.14	0.22 ± 0.13	0.89 ± 0.08	0.27 ± 0.14	0.22 ± 0.13	0.03	0.02	0.00
	NCC-Direct	0.80 ± 0.20	0.74 ± 0.25	0.14 ± 0.13	0.81 ± 0.18	0.80 ± 0.19	0.13 ± 0.12	0.01	0.06	0.01
	NCC-General	0.82 ± 0.07	0.77 ± 0.24	0.09 ± 0.12	0.99 ± 0.01	0.84 ± 0.19	0.06 ± 0.08	0.17	0.07	0.03
	ECC	0.85 ± 0.12	0.81 ± 0.25	0.09 ± 0.12	0.98 ± 0.05	0.95 ± 0.17	0.05 ± 0.09	0.13	0.14	0.04
	MMI	0.85 ± 0.11	0.31 ± 0.19	0.20 ± 0.14	0.86 ± 0.09	0.31 ± 0.16	0.20 ± 0.14	0.01	0.00	0.00
	OF	0.78 ± 0.17	0.56 ± 0.25	0.13 ± 0.12	0.79 ± 0.16	0.61 ± 0.24	0.11 ± 0.09	0.01	0.05	0.02
	TRACER	**0.91** ± 0.10	**0.87** ± 0.19	**0.06** ± 0.10	**1.00** ± 0.00	**1.00** ± 0.00	**0.00** ± 0.00	0.09	0.13	0.06
Sim-seq 6	Unregistered	0.51 ± 0.23	0.42 ± 0.12	0.09 ± 0.10	0.54 ± 0.22	0.44 ± 0.02	0.08 ± 0.05	0.03	0.02	0.01
	NCC-Direct	0.52 ± 0.28	0.57 ± 0.29	0.10 ± 0.09	0.54 ± 0.32	0.56 ± 0.32	0.10 ± 0.08	0.02	-0.01	0.00
	NCC-General	0.53 ± 0.19	0.58 ± 0.18	0.09 ± 0.06	0.56 ± 0.14	0.57 ± 0.17	0.18 ± 0.04	0.03	0.01	0.00
	ECC	0.76 ± 0.26	**0.72** ± 0.30	**0.06** ± 0.08	**0.77** ± 0.21	0.81 ± 0.24	**0.03** ± 0.03	0.01	0.09	0.03
	MMI	0.49 ± 0.20	0.44 ± 0.12	0.09 ± 0.09	0.49 ± 0.16	0.47 ± 0.06	0.08 ± 0.05	0.00	0.03	0.01
	OF	0.49 ± 0.21	0.46 ± 0.15	0.08 ± 0.06	0.52 ± 0.20	0.48 ± 0.09	0.07 ± 0.05	0.03	0.02	0.01
	TRACER	**0.78** ± 0.23	0.71 ± 0.20	**0.06** ± 0.08	0.72 ± 0.29	**0.82** ± 0.18	0.04 ± 0.04	-0.04	0.11	0.02
Sim-seq 7	Unregistered	0.50 ± 0.22	0.39 ± 0.16	0.18 ± 0.17	0.52 ± 0.21	0.41 ± 0.15	0.18 ± 0.17	0.02	0.02	0.00
	NCC-Direct	0.60 ± 0.27	0.59 ± 0.31	0.13 ± 0.13	0.63 ± 0.29	0.55 ± 0.34	0.13 ± 0.13	0.03	-0.04	0.00
	NCC-General	0.62 ± 0.25	0.61 ± 0.18	0.12 ± 0.12	0.65 ± 0.25	0.59 ± 0.17	0.13 ± 0.12	0.02	0.02	0.00
	ECC	0.60 ± 0.27	0.67 ± 0.24	0.11 ± 0.12	0.64 ± 0.27	0.72 ± 0.23	0.11 ± 0.12	0.04	0.05	0.00
	MMI	0.48 ± 0.20	0.38 ± 0.13	0.18 ± 0.16	0.50 ± 0.20	0.40 ± 0.11	0.17 ± 0.16	0.02	0.02	0.01
	OF	0.54 ± 0.22	0.53 ± 0.16	**0.08** ± 0.07	0.56 ± 0.21	0.56 ± 0.14	**0.07** ± 0.05	0.02	0.03	0.01
	TRACER	**0.72** ± 0.34	**0.71** ± 0.25	0.15 ± 0.15	**0.71** ± 0.35	**0.76** ± 0.25	0.15 ± 0.15	-0.01	0.05	0.00

Table III
IMPACT OF STEP 1 ON REGISTRATION METHODS AND TRACER, USING FLIM IMAGE SEQUENCES.

Data	Method	Without Step 1			With Step 1			Metric Δ		
		QA ↑	SSIM ↑	NRMSE ↓	QA ↑	SSIM ↑	NRMSE ↓	QA	SSIM	NRMSE
FLIm-seq 1	Unregistered	0.90 ± 0.04	0.64 ± 0.13	0.14 ± 0.10	0.90 ± 0.04	0.64 ± 0.13	0.14 ± 0.10	-	-	-
	NCC-Direct	0.91 ± 0.06	0.71 ± 0.17	0.13 ± 0.11	0.91 ± 0.06	0.71 ± 0.17	0.13 ± 0.11	-	-	-
	NCC-General	0.91 ± 0.03	0.60 ± 0.12	0.14 ± 0.09	0.91 ± 0.03	0.60 ± 0.12	0.14 ± 0.09	-	-	-
	ECC	0.92 ± 0.03	0.69 ± 0.10	0.13 ± 0.08	0.92 ± 0.03	0.69 ± 0.10	0.13 ± 0.08	-	-	-
	MMI	0.92 ± 0.03	0.69 ± 0.10	0.13 ± 0.09	0.92 ± 0.03	0.69 ± 0.10	0.13 ± 0.09	-	-	-
	OF	0.96 ± 0.02	0.76 ± 0.09	0.08 ± 0.06	0.96 ± 0.02	0.76 ± 0.09	0.08 ± 0.06	-	-	-
	TRACER	**0.98** ± 0.01	**0.91** ± 0.03	**0.06** ± 0.04	**0.98** ± 0.01	**0.91** ± 0.03	**0.06** ± 0.04	-	-	-
FLIm-seq 2	Unregistered	0.92 ± 0.04	0.73 ± 0.06	0.09 ± 0.06	0.92 ± 0.04	0.73 ± 0.06	0.09 ± 0.06	-	-	-
	NCC-Direct	0.93 ± 0.05	0.78 ± 0.11	0.08 ± 0.07	0.93 ± 0.05	0.78 ± 0.11	0.08 ± 0.07	-	-	-
	NCC-General	0.92 ± 0.04	0.76 ± 0.10	0.10 ± 0.08	0.92 ± 0.04	0.76 ± 0.10	0.10 ± 0.08	-	-	-
	ECC	0.92 ± 0.04	0.77 ± 0.06	0.08 ± 0.05	0.92 ± 0.04	0.77 ± 0.06	0.08 ± 0.05	-	-	-
	MMI	0.91 ± 0.04	0.73 ± 0.07	0.09 ± 0.06	0.91 ± 0.04	0.73 ± 0.07	0.09 ± 0.06	-	-	-
	OF	**0.98** ± 0.01	0.82 ± 0.04	0.05 ± 0.03	**0.98** ± 0.01	0.82 ± 0.04	0.05 ± 0.03	-	-	-
	TRACER	**0.98** ± 0.01	**0.94** ± 0.02	**0.04** ± 0.03	**0.98** ± 0.01	**0.94** ± 0.02	**0.04** ± 0.03	-	-	-
FLIm-seq 3	Unregistered	0.68 ± 0.18	0.31 ± 0.11	0.19 ± 0.20	0.75 ± 0.01	0.35 ± 0.02	0.15 ± 0.04	0.07	0.04	0.04
	NCC-Direct	0.74 ± 0.20	0.45 ± 0.25	0.18 ± 0.22	0.81 ± 0.09	0.50 ± 0.22	0.13 ± 0.09	0.07	0.05	0.05
	NCC-General	0.77 ± 0.05	0.38 ± 0.16	0.16 ± 0.10	0.77 ± 0.01	0.33 ± 0.07	0.14 ± 0.06	0.00	-0.05	0.01
	ECC	0.76 ± 0.12	0.36 ± 0.08	0.15 ± 0.15	0.78 ± 0.02	0.38 ± 0.03	0.13 ± 0.05	0.02	0.02	0.02
	MMI	0.71 ± 0.19	0.29 ± 0.11	0.18 ± 0.20	0.78 ± 0.02	0.34 ± 0.06	0.14 ± 0.06	0.07	0.05	0.04
	OF	**0.79** ± 0.17	0.47 ± 0.11	**0.15** ± 0.17	0.85 ± 0.01	0.51 ± 0.02	0.11 ± 0.03	0.06	0.04	0.04
	TRACER	0.78 ± 0.18	**0.53** ± 0.25	0.15 ± 0.18	**0.92** ± 0.00	**0.83** ± 0.01	**0.09** ± 0.02	0.14	0.30	0.06
FLIm-seq 4	Unregistered	0.55 ± 0.19	0.38 ± 0.15	0.23 ± 0.19	0.58 ± 0.16	0.40 ± 0.11	0.21 ± 0.14	0.03	0.02	0.02
	NCC-Direct	0.66 ± 0.21	0.52 ± 0.23	0.20 ± 0.16	0.67 ± 0.21	0.54 ± 0.23	0.19 ± 0.16	0.01	0.02	0.01
	NCC-General	0.71 ± 0.07	0.53 ± 0.17	0.20 ± 0.11	0.72 ± 0.07	0.51 ± 0.16	0.19 ± 0.10	-0.01	-0.02	0.00
	ECC	0.58 ± 0.16	0.39 ± 0.12	0.21 ± 0.15	0.59 ± 0.16	0.39 ± 0.11	0.21 ± 0.13	0.01	0.00	0.00
	MMI	0.55 ± 0.19	0.31 ± 0.14	0.23 ± 0.18	0.58 ± 0.16	0.35 ± 0.12	0.22 ± 0.14	0.03	0.04	0.01
	OF	**0.82** ± 0.12	0.54 ± 0.11	**0.13** ± 0.11	**0.85** ± 0.04	0.56 ± 0.08	**0.12** ± 0.06	0.03	0.02	0.01
	TRACER	0.69 ± 0.21	**0.56** ± 0.21	0.19 ± 0.16	0.79 ± 0.03	**0.57** ± 0.05	0.13 ± 0.05	0.10	0.01	0.06
FLIm-seq 5	Unregistered	0.62 ± 0.23	0.33 ± 0.22	0.25 ± 0.21	0.63 ± 0.22	0.33 ± 0.22	0.24 ± 0.21	0.01	0.00	0.01
	NCC-Direct	0.77 ± 0.14	0.49 ± 0.23	0.18 ± 0.14	0.77 ± 0.14	0.50 ± 0.23	0.18 ± 0.14	0.00	0.01	0.00
	NCC-General	0.78 ± 0.09	0.55 ± 0.22	0.17 ± 0.11	0.87 ± 0.09	0.55 ± 0.22	0.17 ± 0.11	0.00	-0.01	0.00
	ECC	0.71 ± 0.14	0.43 ± 0.14	0.22 ± 0.18	0.72 ± 0.14	0.43 ± 0.14	0.22 ± 0.18	0.01	0.00	0.00
	MMI	0.64 ± 0.23	0.33 ± 0.20	0.24 ± 0.20	0.65 ± 0.23	0.33 ± 0.20	0.23 ± 0.20	0.01	0.00	0.01
	OF	**0.86** ± 0.11	0.53 ± 0.17	**0.13** ± 0.11	**0.87** ± 0.10	0.53 ± 0.16	0.13 ± 0.11	0.01	0.00	0.00
	TRACER	0.78 ± 0.09	**0.55** ± 0.14	0.15 ± 0.13	0.83 ± 0.02	**0.61** ± 0.06	**0.13** ± 0.05	0.05	0.10	0.02
FLIm-seq 6	Unregistered	0.62 ± 0.21	0.33 ± 0.16	0.33 ± 0.26	0.65 ± 0.21	0.36 ± 0.13	0.32 ± 0.26	0.03	0.03	0.01
	NCC-Direct	0.73 ± 0.23	0.53 ± 0.24	0.28 ± 0.26	0.77 ± 0.23	0.59 ± 0.22	0.26 ± 0.26	0.04	0.06	0.02
	NCC-General	0.72 ± 0.05	0.62 ± 0.19	0.18 ± 0.13	0.78 ± 0.04	0.62 ± 0.19	0.17 ± 0.10	0.02	0.00	0.00
	ECC	0.73 ± 0.17	0.47 ± 0.09	0.27 ± 0.21	0.73 ± 0.17	0.48 ± 0.08	0.27 ± 0.21	0.00	0.01	0.00
	MMI	0.67 ± 0.18	0.39 ± 0.13	0.29 ± 0.21	0.70 ± 0.16	0.41 ± 0.10	0.28 ± 0.21	0.03	0.02	0.01
	OF	**0.89** ± 0.09	0.56 ± 0.13	0.17 ± 0.16	**0.92** ± 0.05	0.60 ± 0.06	0.15 ± 0.12	0.03	0.04	0.02
	TRACER	0.77 ± 0.11	**0.68** ± 0.17	**0.15** ± 0.18	0.79 ± 0.02	**0.69** ± 0.05	**0.13** ± 0.10	0.02	0.01	0.02

Table IV
IMPACT OF STEP 2 ON REGISTRATION METHODS AND TRACER, USING SIMULATION IMAGE SEQUENCES.

Data	Method	After step 1, without step 2			After step 1 and 2			Metric Δ		
		QA ↑	SSIM ↑	NRMSE ↓	QA ↑	SSIM ↑	NRMSE ↓	QA	SSIM	NRMSE
Sim-seq 6	Unregistered	0.54 ± 0.22	0.44 ± 0.02	0.08 ± 0.05	0.72 ± 0.18	0.52 ± 0.05	0.08 ± 0.04	0.18	0.08	0.00
	NCC-Direct	0.54 ± 0.32	0.56 ± 0.32	0.10 ± 0.08	0.78 ± 0.29	0.78 ± 0.29	0.06 ± 0.08	0.24	0.22	0.04
	NCC-General	0.61 ± 0.14	0.68 ± 0.17	0.07 ± 0.04	0.86 ± 0.10	0.74 ± 0.17	0.05 ± 0.04	0.00	-0.04	-0.01
	ECC	**0.83** ± 0.21	0.81 ± 0.24	**0.03** ± 0.03	0.89 ± 0.16	0.88 ± 0.17	0.03 ± 0.04	0.06	0.07	0.00
	MMI	0.49 ± 0.16	0.47 ± 0.06	0.08 ± 0.05	0.66 ± 0.16	0.54 ± 0.08	0.08 ± 0.06	0.17	0.07	0.00
	OF	0.52 ± 0.20	0.48 ± 0.09	0.07 ± 0.05	0.70 ± 0.18	0.60 ± 0.12	0.06 ± 0.04	0.18	0.12	0.01
	TRACER	0.72 ± 0.29	**0.82** ± 0.18	0.04 ± 0.04	**0.98** ± 0.05	**0.98** ± 0.04	**0.01** ± 0.02	0.26	0.16	0.03
Sim-seq 7	Unregistered	0.52 ± 0.21	0.41 ± 0.15	0.18 ± 0.17	0.70 ± 0.18	0.39 ± 0.14	0.16 ± 0.10	0.18	-0.02	0.02
	NCC-Direct	0.63 ± 0.29	0.55 ± 0.34	0.13 ± 0.13	0.84 ± 0.22	0.78 ± 0.28	0.09 ± 0.11	0.21	0.23	0.04
	NCC-General	0.63 ± 0.25	0.65 ± 0.17	0.15 ± 0.12	0.94 ± 0.10	0.86 ± 0.20	0.07 ± 0.08	0.20	0.08	0.04
	ECC	0.64 ± 0.27	0.72 ± 0.23	0.11 ± 0.12	0.88 ± 0.16	0.86 ± 0.22	0.06 ± 0.08	0.24	0.14	0.05
	MMI	0.50 ± 0.20	0.40 ± 0.11	0.17 ± 0.16	0.67 ± 0.18	0.40 ± 0.13	0.16 ± 0.11	0.17	0.00	0.01
	OF	0.56 ± 0.21	0.56 ± 0.14	0.07 ± 0.05	0.67 ± 0.19	0.58 ± 0.19	0.10 ± 0.08	0.11	0.02	-0.03
	TRACER	**0.65** ± 0.35	**0.76** ± 0.25	**0.12** ± 0.15	**0.98** ± 0.06	**0.97** ± 0.05	**0.03** ± 0.04	0.33	0.21	0.12

Table V
IMPACT OF STEP 2 ON REGISTRATION METHODS AND TRACER, USING FLIM IMAGE SEQUENCES.

Data	Method	After step 1, without step 2			After step 1 and 2			Metric Δ		
		QA ↑	SSIM ↑	NRMSE ↓	QA ↑	SSIM ↑	NRMSE ↓	QA	SSIM	NRMSE
FLIm-seq 4	Unregistered	0.58 ± 0.16	0.40 ± 0.11	0.21 ± 0.14	0.60 ± 0.11	0.40 ± 0.09	0.20 ± 0.11	0.02	0.00	0.01
	NCC-Direct	0.67 ± 0.21	0.54 ± 0.23	0.19 ± 0.16	0.76 ± 0.16	0.66 ± 0.17	0.14 ± 0.12	0.09	0.12	0.05
	NCC-General	0.72 ± 0.07	0.51 ± 0.16	0.19 ± 0.10	0.76 ± 0.07	0.50 ± 0.16	0.18 ± 0.10	0.04	-0.01	0.01
	ECC	0.59 ± 0.16	0.39 ± 0.11	0.21 ± 0.13	0.64 ± 0.11	0.37 ± 0.11	0.19 ± 0.10	0.05	-0.02	0.02
	MMI	0.58 ± 0.16	0.35 ± 0.12	0.22 ± 0.14	0.60 ± 0.11	0.34 ± 0.11	0.20 ± 0.11	0.02	-0.01	0.02
	OF	**0.85** ± 0.04	0.56 ± 0.08	**0.12** ± 0.06	0.86 ± 0.03	0.56 ± 0.07	0.12 ± 0.06	0.01	0.00	0.00
	TRACER	0.79 ± 0.03	**0.57** ± 0.05	0.13 ± 0.05	**0.93** ± 0.02	**0.82** ± 0.04	**0.08** ± 0.04	0.14	0.25	0.05
FLIm-seq 5	Unregistered	0.63 ± 0.22	0.38 ± 0.22	0.24 ± 0.21	0.94 ± 0.02	0.75 ± 0.04	0.08 ± 0.05	0.31	0.37	0.16
	NCC-Direct	0.77 ± 0.14	0.50 ± 0.23	0.18 ± 0.14	0.97 ± 0.02	0.89 ± 0.07	0.06 ± 0.05	0.20	0.39	0.12
	NCC-General	0.77 ± 0.09	0.58 ± 0.22	0.17 ± 0.11	0.94 ± 0.01	0.71 ± 0.07	0.10 ± 0.06	0.10	0.11	0.05
	ECC	0.72 ± 0.14	0.43 ± 0.14	0.22 ± 0.18	0.95 ± 0.01	0.78 ± 0.04	0.08 ± 0.04	0.23	0.35	0.14
	MMI	0.65 ± 0.23	0.37 ± 0.20	0.23 ± 0.20	0.95 ± 0.01	0.73 ± 0.06	0.09 ± 0.05	0.30	0.36	0.14
	OF	**0.87** ± 0.10	0.53 ± 0.16	**0.13** ± 0.11	0.97 ± 0.01	0.85 ± 0.03	0.05 ± 0.03	0.10	0.32	0.08
	TRACER	0.83 ± 0.02	**0.61** ± 0.06	0.17 ± 0.05	**0.98** ± 0.01	**0.89** ± 0.01	**0.05** ± 0.02	0.15	0.28	0.12
FLIm-seq 6	Unregistered	0.65 ± 0.21	0.36 ± 0.13	0.32 ± 0.26	0.78 ± 0.09	0.47 ± 0.10	0.20 ± 0.12	0.13	0.11	0.12
	NCC-Direct	0.77 ± 0.23	0.59 ± 0.22	0.26 ± 0.26	0.89 ± 0.06	0.79 ± 0.08	0.15 ± 0.09	0.12	0.20	0.11
	NCC-General	0.79 ± 0.04	0.68 ± 0.19	0.15 ± 0.10	0.91 ± 0.04	0.65 ± 0.11	0.14 ± 0.09	0.12	-0.03	0.01
	ECC	0.73 ± 0.17	0.48 ± 0.08	0.27 ± 0.21	0.83 ± 0.08	0.54 ± 0.08	0.17 ± 0.11	0.10	0.06	0.10
	MMI	0.70 ± 0.16	0.41 ± 0.10	0.28 ± 0.21	0.80 ± 0.08	0.46 ± 0.08	0.19 ± 0.12	0.10	0.05	0.09
	OF	**0.92** ± 0.05	0.60 ± 0.06	0.15 ± 0.12	0.94 ± 0.01	0.68 ± 0.05	**0.09** ± 0.05	0.02	0.08	0.06
	TRACER	0.79 ± 0.02	**0.69** ± 0.05	**0.13** ± 0.10	**0.95** ± 0.02	**0.85** ± 0.04	**0.09** ± 0.05	0.16	0.16	0.04

Table VI
PAIRED T-TEST RESULTS COMPARING REGISTRATION METHODS BEFORE AND AFTER APPLYING STEP 1 AND 2. SIGNIFICANT DIFFERENCES ($p < 0.05$) INDICATED WITH *.

Method	Before using the proposed pipeline			After Steps 1 & 2			Paired t-test results		
	QA ↑	SSIM ↑	NRMSE ↓	QA ↑	SSIM ↑	NRMSE ↓	QA (p)	SSIM (p)	NRMSE (p)
Unregistered	0.513	0.374	0.173	0.781	0.539	0.135	0.012*	0.012*	0.012*
NCC-Direct	0.701	0.536	0.204	0.792	0.761	0.113	0.411	0.411	0.411
NCC-General	0.704	0.516	0.243	0.916	0.639	0.166	0.000*	0.000*	0.000*
ECC	0.571	0.410	0.217	0.780	0.548	0.198	0.015*	0.015*	0.015*
MMI	0.547	0.207	0.342	0.803	0.511	0.280	0.012*	0.012*	0.012*
OF	0.839	0.566	0.178	0.943	0.678	0.059	0.005*	0.005*	0.005*
TRACER	0.668	0.506	0.157	0.940	0.850	0.092	0.001*	0.001*	0.001*

Table VII
STATISTICAL COMPARISON BETWEEN TRACER AND OTHER REGISTRATION METHODS FOR PRECISION, RECALL, AND F1-SCORE.

Method	Precision		Recall		F1-Score	
	p-value	Significance	p-value	Significance	p-value	Significance
Unregistered	0.547	False	0.000	True	0.000	True
NCC-Translate	0.004	True	0.000	True	0.000	True
NCC-General	0.248	False	0.000	True	0.000	True
ECC	0.276	False	0.000	True	0.000	True
MMI	0.030	True	0.000	True	0.000	True
OF	0.000	True	0.000	True	0.000	True
